# USP18 Impacts Cisplatin Resistance in Ovarian Cancer Cells by Modulating DNA Repair

**DOI:** 10.7150/ijbs.130399

**Published:** 2026-05-29

**Authors:** Cristina Corno, Matteo Costantino, Pietro Pettinari, Luca Mirra, Simone Stucchi, Noemi Arrighetti, Nunzio Perta, Giovanni Di Muccio, Mathilde Robin, Giovanni L. Beretta, Elisabetta Corna, Nives Carenini, Loredana Cleris, Diego Colombo, Elena Luison, Chiara Maura Ciniselli, Mara Lecchi, Paolo Verderio, Mariangela Figini, Stig Linder, Diego Tosi, Anna La Teana, Padraig D'Arcy, Paola Perego

**Affiliations:** 1 Molecular Pharmacology Unit, Department of Experimental Oncology, Fondazione IRCCS Istituto Nazionale dei Tumori, via Amadeo 42, 20133 Milan, Italy.; 2 Polytechnic University of Marche, Department of Life and Environmental Science, via Brecce Bianche, 60131 Ancona, Italy.; 3 Early Clinical Trial Unit, Medical Oncology Department, Institut régional du Cancer Montpellier, Inserm U1194, Montpellier University, France.; 4 Department of Medical Biotechnology and Translational Medicine, University of Milan, via Saldini 50, 20133 Milan, Italy.; 5 Department of Advanced Diagnostics, Fondazione IRCCS Istituto Nazionale dei Tumori, via Amadeo 42, 20133 Milano, Italy.; 6 Unit of Bioinformatics and Biostatistics, Department of Epidemiology and Data Science, Fondazione IRCCS Istituto Nazionale dei Tumori, via Venezian 1, 20133 Milan, Italy.; 7 Department of Biomedical and Clinical Sciences (BKV), SE-581 83, Linköping, Sweden.; 8 Division of Biochemistry, Department of Medical Biochemistry and Biophysics, Karolinska Institutet, Stockholm, Sweden.; 9 Fondazione Gianni Bonadonna, via Bertani 14, 20154 Milan, Italy.

**Keywords:** deubiquitinases, cisplatin, drug-resistance, ovarian carcinoma, CRISPR-Cas9, USP18

## Abstract

Deubiquitinases (DUBs) are proteases with emerging roles in cancer, yet their contribution to drug resistance in ovarian cancer remains underexplored. Ovarian cancer patients often fail to benefit from platinum-based therapy, highlighting the need to identify novel factors driving drug resistance. Thus, we performed a CRISPR/Cas9 screen targeting the DUB family to identify genes essential for cisplatin-resistant ovarian carcinoma cell survival. CRISPR/Cas9 DUB knockout screens, preclinical pharmacology approaches, RNA sequencing, proteomic analyses, computational tools, surface plasma resonance were applied. We identified USP18 as a survival factor in cisplatin-resistant ovarian cancer cells. USP18 expression was elevated at the mRNA and protein levels across five cisplatin-resistant variants. Knockdown and CRISPR/Cas9 editing of USP18 sensitized cells to cisplatin, coinciding with impaired repair of cisplatin-induced DNA damage. Enhanced sensitivity to cisplatin was evident from studies in mice. RNA-seq of USP18 RNA interfered and edited cells revealed the modulation of pathways including DNA repair. A peptide-based USP18 inhibitor suppressed growth of cisplatin-resistant cells, supporting USP18 role in sustaining their growth. We identified USP18 as a novel mediator of cisplatin resistance in ovarian cancer, acting through DNA repair modulation. Targeting USP18 may offer a therapeutic strategy to improve outcomes in platinum-resistant ovarian cancer.

## Introduction

Ovarian carcinoma is a heterogeneous disease that accounts for the majority of deaths from gynecological cancers 1. Despite advances in the understanding of tumor molecular landscapes and pharmacological interventions, platinum-based therapy remains the standard of care for this disease 2. Both cisplatin and carboplatin are used in ovarian carcinoma treatment, with carboplatin showing a more tolerable profile. The DNA adducts produced by carboplatin and cisplatin are identical 3 resulting in overlapping cell responses to both drugs. Although a few targeted therapies, such as bevacizumab, PARP inhibitors and mirvetuximab soravtansine 4 are available, drug resistance remains a major clinical obstacle for most patients. Platinum drug resistance is a complex phenomenon involving multiple cellular mechanisms 5. These include reduced drug accumulation, increased detoxification and efflux, and altered DNA repair mechanisms. Altered thiol metabolism has been shown to impair cisplatin accumulation, with cancer-associated fibroblasts contributing to reduced nuclear platinum uptake 6. Early studies postulated roles for transporters, gated channels, and passive diffusion in platinum drug uptake 7,8 and subsequent work established that VRAC channels mediate cisplatin accumulation 9. Restoration of DNA repair has been linked to resistance as shown by secondary BRCA1 mutations that restore the wild-type gene reading frame in ovarian carcinomas with primary or acquired cisplatin resistance 10. Similarly, restoration of BRCA2 expression has also been reported as a mechanism of cisplatin resistance in BRCA2 mutant breast and pancreatic cancer preclinical models 11. Despite these advances in understanding platinum resistance, clinical translation has remained limited, and there is a continued need to identify new targetable mechanisms. Emerging evidence shows that deubiquitinases (DUBs) 12, enzymes that remove ubiquitin chains from target proteins, play a role as regulators of tumor cell survival, DNA repair and apoptosis 13. For instance, USP1 promotes ovarian carcinoma progression by stabilizing DNA damage response proteins 14, and inhibitors of USP1 have entered early clinical development. We have also previously implicated USP8, a DUB linked to growth factor signaling, in cisplatin-resistant ovarian cancer models and found that USP8 is an independent predictor of poor prognosis 15. However, only 40% of clinical specimens expressed USP8, implying that other factors including additional DUBs participate in drug resistance. Given the molecular heterogeneity of ovarian cancer, a broader functional screen was warranted to identify other DUBs involved in platinum resistance.

Based on this background, the aim of this study was to identify DUBs essential for the survival of cisplatin-resistant cells using a CRISPR/Cas9 dropout screen and to validate the role of the identified DUBs by functional studies in ovarian carcinoma cells of various histotypes. Through this approach, we identified Ubiquitin-specific protease 18 (USP18), the main ISG15 (Interferon-stimulated gene product 15) deconjugating enzyme, as a gene capable of conferring increased survival to platinum-resistant cells. After identification of key DUBs by CRISPR/Cas9 drop out screen, we used molecular inactivation by knock-down upon siRNA transfection and gene editing by CRISPR/Cas9 together with RNA sequencing and global proteomics to identify key molecular players associated with the phenotypes observed in engineered resistant cells. Furthermore, through molecular modeling we designed peptides capable of inhibiting USP18.

## Material and Methods

### Cell lines and cell sensitivity assays

A panel of human ovarian carcinoma cell lines was used for this study, including pairs of cisplatin-sensitive (IGROV-1, OVCAR5, A2780 and TOV21G) and -resistant cells (IGROV-1/Pt1, OVCAR5/Pt, A2780/CP and TOV21G/Pt), as well as Caov3, TOV112D, ES-2 and SKOV-3 purchased from ATCC (Washington, DC, NY); PEO1 and PEO4 cell lines derived from a high-grade serous ovarian cancer patient before and after development of platinum resistance, purchased from Sigma-Aldrich (Milan, Italy); OVCAR3, OVCAR4, OVCAR8 cells obtained from the DCTD tumor repository (NCI, Frederick MD, USA). Doxorubicin-resistant IGROV-1/DX 16 and oxaliplatin-resistant IGROV-1/OHP 17 cells were obtained from IGROV-1 by exposure to increasing drug concentrations. All cell lines were grown in RPMI-1640 medium (Gibco, Thermo Fisher, Monza, Italy) supplemented with 10% FBS (Euroclone, S. p. A., Pero, Italy), except for Caov3 cultured in DMEM medium (Sigma-Aldrich) and ES-2 cells cultured in McCoy's 5A (Euroclone). Cells were cultured for no more than 20 passages and routinely checked for mycoplasma using MycoAlert kit (Lonza, Basel, Switzerland). The effect of cisplatin was evaluated using colony forming assays in soft-agar. Briefly, cells were treated with different concentrations of drug for 1 h and then seeded into 35 mm dishes (2500 cells/cm^2^) in 0.33% agarose on 0.5% agarose bed and incubated for around two weeks. Cells were stained with p-iodonitrotetrazolium violet (Sigma-Aldrich) 1 mg/ml in saline and 24 h later colonies of at least 50 cells were counted under a magnifying projector. IC_50_ values refer to the concentration required to reduce colony formation by 50%. IGROV-1/Pt1 cell sensitivity to USP18 peptides was measured by growth inhibition assays or MTS assays (see Supplementary Material and Methods); cells were seeded in 12-well plates and then treated for 24 h with different concentration of peptides in serum-free medium; also control samples were performed under identical serum-free conditions, ensuring that any observed effect was specific to the USP18 peptides. Fresh completed medium was then added for 48 h. Seventy-two hours after treatment start, cells were counted with a cell counter (Beckman Coulter, Milan, Italy). IC_50_ values represent the drug concentrations producing 50% cell number decrease. The peptides were purchased from Covalab (Bron, France). The USP18-targeting peptides were synthetized by solid phase peptide synthesis and their purity were greater than 95%, as indicated in the datasheet. Cisplatin (Sandoz, Origgio, Italy) was diluted in saline; USP18-derived peptides were diluted in distilled water.

### CRISPR/Cas9 dropout screen

Stable Cas9-expressing IGROV-1, IGROV-1/Pt1, PEO1, and PEO4 cells were generated by lentiviral transduction with pLenti-Cas9-T2A-Blast-BFP, encoding a codon-optimized WT SpCas9 fused via a self-cleaving peptide to blasticidin-S-deaminase and mTagBFP. Following selection with 2 µg/mL blasticidin, stable BFP^+^ populations were established. For the CRISPR dropout screen, viral titers of the guide RNA (gRNA) library were determined, and Cas9-BFP-expressing cells were transduced in duplicate at a multiplicity of infection of 0.3, maintaining a 1,000x coverage (1000 cells per gRNA). A custom pooled sgRNA library was generated, targeting 87 DUBs, along with essential proteasome subunits as positive controls and olfactory receptor genes as negative cutting controls. Puromycin selection was applied from day 2 to day 6 post-transduction. Throughout the screen, cell numbers were maintained at ≥80 million per replicate. A control sample of 80 million cells per replicate was harvested on day 4. Genomic DNA was extracted after 21 days, and guide sequences were amplified and subjected to Illumina NovaSeq sequencing. Next-generation sequencing data were analyzed using MaGeCK software and unique molecular identifier lineage dropout analysis for comprehensive evaluation of gene essentiality.

### Quantitative Real Time PCR

Gene expression was evaluated in ovarian carcinoma cell lines and ovarian tumor specimens using quantitative RT-PCR (qRT-PCR). Total RNA was isolated using RNeasy Plus Mini kit (Qiagen, Hilden, Germany) and retrotranscribed with the High Capacity cDNA Reverse Transcription kit (Applied Biosystems, Thermo Fisher), according to manufacturer's protocol. Reactions (5 µl TaqMan Fast mix, 0.5 µl specific Taqman assay, 2.5 µl cDNA) were carried out in triplicate using the QuantStudio 7 Flex Instrument (Thermo Fisher Scientific, Waltham, Massachusetts, USA); for analysis of results QuantStudio 6 and 7 Flex software were used. In this study, the following TaqMan assays were used: USP18_Hs07289021_m1; GAPDH_Hs99999905_m1; RPS13_Hs01011488_g1. The determination of target gene levels was performed using the relative quantification method (RQ) based on the comparative Ct calculation: ΔCt = Ct gene - Ct housekeeping gene; ΔΔCt = ΔCt sample - ΔCt calibrator; RQ = 2^- ΔΔCt^; GAPDH or RPS13 were used as housekeeping genes; untransfected cells were used as calibrator for siRNA transfection.

### Western blot analysis

For western blot analysis, cells were lysed according to a standard protocol. The lysis buffer consisted of 0.125 M Tris HCl pH 6.8, 5% SDS (Lonza) and protease inhibitors (25 mM sodium fluoride, 10 μg/ml pepstatin A, 1 mM phenylmethylsulfonyl fluoride, 10 μg/ml trypsin inhibitor, 12.5 μg/ml leupeptin, 30 μg/ml aprotinin, 1 mM sodium orthovanadate and 1 mM sodium molybdate, all purchased from Sigma-Aldrich). Samples were boiled for 5 min and sonicated for 25 sec. Quantification of protein lysates was carried out with the BCA method (Pierce, Thermo Fisher Scientific). Proteins were fractionated by SDS-PAGE and transferred to nitrocellulose membranes. Blots were blocked in PBS with 5% dried non-fat milk and incubated overnight at 4°C with primary antibodies. Chemiluminescence detection of immunoreactive bands was achieved using ECL (GE Healthcare, Milan, Italy) upon exposure to films or by Azure 600 imager (Azure Biosystems, Aurogene s.r.l., Rome, Italy). The band intensities were quantified using ImageJ 1.47v software. The following antibodies were used according to manufacturer's instructions: anti-actin and anti-vinculin from Sigma-Aldrich; anti-β tubulin from Abcam (Cambridge, UK); anti-RAD50 and anti-XPC from Invitrogen (Thermo Fisher); anti-USP18 from Cell Signaling (Danvers, Massachusetts, USA). Amersham ECL anti-mouse/rabbit peroxidized secondary antibody (GE Healthcare). The antibody details are reported in Supplementary Table S1.

### Public database and clinical samples

To study the relationship between USP18 and patient survival, we used the dataset of the Cancer Genome Atlas (TCGA) Research network 18. In addition, we carried out a retrospective analysis using a case material comprising clinical specimens from ovarian cancer patients described previously 19. Consent for the use of clinical information for research was obtained for all patients. The study was approved from the review board of the Fondazione IRCCS Istituto Nazionale dei Tumori (protocol INT 23/21). Briefly, the cohort included 134 ovarian carcinoma patients (Supplementary Table S2) 19. USP18 expression was considered in relation to GAPDH and RPS13 as housekeeping genes taken singly and in combination in terms of log_2_RQ (i.e., -ΔCt).

### Loss of function studies and generation of CRISPR/Cas9 knock-out clones

USP18 gene molecular inactivation was achieved using two different approaches: 1) RNA interference by transfection with small interfering RNAs (siRNAs) mediated by lipofectamine RNAiMAX in OptiMEM medium; 2) generation of stable knockout (KO) clones through the CRISPR/Cas9 method. Different siRNAs directed against USP18 [Silencer_Select s22259 (siRNA a), s22260 (siRNA b) and s22261 (siRNA c), Thermo Fisher] or Silence Select Negative Control #2 siRNA (Thermo Fisher Scientific) were used. Twenty-four h after seeding in 6-well plates, IGROV-1/Pt1 cells were transfected with 10 nM siRNAs for 5 h; after that time, transfection medium was replaced with complete medium. USP18 levels were evaluated by qRT-PCR and, 48 h after transfection start, cells were reseeded for: 1) colony forming assay in soft-agar; 2) modified COMET assay.

For generation of stable KO clones, equimolar mixtures of two synthetic single-guide RNAs (sgRNAs) targeting USP18 (ENST00000215794.8) were used: caggccctgggacgctctct and gcaaatctgtcagtccatcc (CRISPRevolution sgRNA EZ Kit, Synthego, Redwood City, CA, USA). The first coding exon was targeted due to high sequence homology between USP18 and USP41 in downstream exons, which made the design of other exon-specific sgRNAs infeasible. sgRNAs were pre-complexed with recombinant Cas9 protein (Alt-R S.p. Cas9 Nuclease V3, Integrated DNA Technologies, IDT, Cornelville, Iowa, USA) to form ribonucleoprotein complexes. These were delivered into 2.5 × 10⁵ cells per sample via electroporation using the Neon Transfection System (Thermo Fisher) with a single 30 ms pulse at 1400 V and a 100 µL tip. Following electroporation, cells were expanded for several days before genomic DNA was extracted using the QIAamp DNA Mini Kit (Qiagen). The frequency of disrupted alleles was estimated by droplet digital PCR (ddPCR) using the QX200 System (Bio-Rad, Hercules, CA, USA) in a drop-off assay format, employing a HEX-labeled reference probe and a FAM-labeled drop-off probe (IDT), the latter designed to detect the disrupted target site corresponding to one of the sgRNAs. Two KO clones, i.e., clone 2 and clone 14 were used in the present study. Stable wild-type clones (designated as C7 and E10) were generated by the CRISPR/Cas9 approach using Alt-R Cas9 Control kit Human (IDT).

To verify the occurrence of gene editing, genomic DNA was extracted from two IGROV-1/Pt1 derived clones, i.e., clone 2 KO and clone 14 KO (PureLink™ Genomic DNA, Thermo Fisher) following the manufacturer's instructions. Briefly, cells were harvested, lysed, and DNA was purified using spin columns and eluted in nuclease-free water. DNA concentration and purity were assessed using a NanoDrop spectrophotometer (Thermo Scientific). The target genomic region encompassing the CRISPR/Cas9 editing site was amplified by PCR (GoTaq® G2 Flexi DNA Polymerase, Promega, Madison, Wisconsin, USA) using specific primers flanking the expected deletion site (FW: gctctttggcatcagaacgg and REV: aaactcaggcacaggaaagc). PCR products, which resulted in two bands of 479 and 381 bp for both the clones, were agarose-gel purified (PureLink™ Quick Gel Extraction Kit, Thermo Fisher) and submitted for Sanger sequencing (Eurofin, Milan, Italy). Sequencing results were analyzed by alignment to the reference sequence to confirm the presence and precise nature of the CRISPR/Cas9-induced deletion (Supplementary Figure S1).

### Quantitative analysis of DNA damage

A modified COMET assay, in which the gels were treated with a fixed amount of H_2_O_2_ for 5 min before adding a lysis solution to induce DNA breaks, was used to detect cisplatin-induced cross-links as described 20. DNA breaks artificially induced by adding hydrogen peroxide to the samples are reduced by the cisplatin cross-linking ability. DNA breaks were monitored using single-cell gel electrophoresis (CometAssay kit, Trevigen, Helgerman Court Gaithersburg, MD, USA), performed according to the manufacturer's instructions. Briefly, cells were treated with cisplatin for 1 h, embedded in a low melting point agarose at 37 °C after 24 h of recovery in drug-free medium, placed on slides and left to solidify. They were then immersed for 30 min in a lysis solution in the dark and subjected to horizontal gel electrophoresis in alkaline buffer for 10 min. After electrophoresis, the slides were neutralized, immersed for 5 min in 70% ethanol and at last air-dried and scored for DNA migration. Prior to scoring, the DNA was stained using Syber Green. The samples were examined with a fluorescent microscope (Leica Microsystems, Milan, Italy) equipped with a digital camera and a specific software (Comet Assay IV Image Analysis System, Perceptive Instruments, UK). The Olive tail moment (OTM) was used to evaluate DNA damage as described 21. At least 50 cells were scored for each sample in triplicate in at least 2 independent experiments. The cross-linking capability of cisplatin i.e., cisplatin-induced damage could be assessed by the reduction in the tail moment. A cross-link index was built by subtracting the mean tail moment of cisplatin and H_2_O_2_ treated samples from H_2_O_2_ treated samples. A decrease of this index indicates enhanced damage.

### RNA sequencing

For RNA-seq analysis, RNA was extracted using miRNeasy mini kit and sample quality was checked using Tape Station (Agilent Technologies, Santa Clara, California, USA). Samples with RIN > 7 were used. The mRNA sequencing experiments were carried out at Plateforme de Genotypage/Sequençage service (Institute du Cerveau/ Paris Brain Institute, ICM, Paris), using Illumina Stranded mRNA Prep. Raw RNA-seq reads were subjected to quality control using FastQC (v0.11.9) 22 and preprocessing using fastp (v0.23.1) 23. Cleaned reads were aligned to the human reference genome (GRCh38/hg38) using the STAR aligner (v2.7.10b) 24. Gene-level quantification was performed using feature Counts from the Subread package (v2.0.1) 25, which summarizes aligned reads according to gene annotations corresponding to GRCh38/hg38, providing a matrix of raw counts for each gene across all samples. Differential gene expression analysis was conducted using the limma-voom pipeline 26. Low-expressed genes were filtered out. Library sizes were normalized using the trimmed mean of M-values (TMM) method implemented in the edgeR package (v4.0.16) 27. The voom transformation was applied to model the mean-variance relationship 28. Linear models were fitted to the transformed data using the limma package (v3.58.1) 26 with empirical Bayes moderated t-statistics. Gene Set Enrichment Analysis (GSEA) 29 was performed using the fgsea package (v1.28.0) 30 against the Molecular Signatures Database (MSigDB; v2024.1.Hs) 31 to identify significantly enriched biological pathways and processes. Adjusted p-values for multiple testing were calculated with the Benjamini-Hochberg procedure to control the false discovery rate (FDR). All statistical analyses were performed in R (v4.3.1) 32. The RNA-seq data are available in a public repository (https://www.ncbi.nlm.nih.gov/geo/query/acc.cgi?acc=GSE326132).

### Antitumor activity studies

Female Athymic Nude-Foxn1nu mice 6-7 weeks-old (Envigo, Udine, Italy) were employed. Mice were maintained in laminar flow rooms keeping temperature and humidity constant. Mice had free access to food and water. Experiments were authorized by the Italian Ministry of Health according to the national law (Project n° 528/2024-PR) in compliance with international policies and guidelines. The IGROV-1/Pt1 and the derived control and USP18 KO clones were used in the study. Exponentially growing cells (10x10^6^/mouse) were s.c. injected into the right flank on day 0 forming experimental groups of 6 animals. Tumor growth was followed by biweekly measurements of tumor diameters with a Vernier caliper. Tumor volume (TV) was calculated according to the formula: TV (mm^3^) = d^2^xD/2 where d and D are the shortest and the longest diameter, respectively. Cages were randomly assigned one of the five inocula, and subsequently, the two treatments were randomized across cages, balancing for the mean body weight and tumor volume, respectively. Treatment, starting 5 days after cell inoculum, was with cisplatin at 4.5 mg/kg, i.v. every week for 3 weeks. Cisplatin (Sandoz) was diluted in saline and was delivered in a volume of 10 ml/kg of body weight. Tumor volume inhibition percentage (TVI %) in treated versus control mice was calculated as: TVI% = 100-(mean TV treated/mean TV control x 100).

### Proteomic analysis

IGROV-1/Pt1 clone 14 KO was transduced with UBP43 (USP18, NM_017414) human tagged ORF clone lentiviral particles or empty vector with a puromycin cassette (Tema Ricerca, Milan, Italy). Briefly, cells were seeded in 24-well plates (300.000 cells/well) and 24 h later incubated for 24 h with the particles according to the manufacturer's protocol. Selection with 5 µg/ml puromycin started 144 h later. Proteomic analysis was carried out in transduced cells, following immune-precipitation of the DDK tagged protein as described in Supplementary Material and Methods.

For proteomics, all samples were analyzed at UNITECH OMICs (University of Milan, Italy) using: Dionex Ultimate 3000 nano-LC system (Sunnyvale CA, USA) connected to an Orbitrap Exploris™ 240 or to an Orbitrap Fusion™ Tribrid™ Mass Spectrometer (Thermo Scientific, Bremen, Germany) equipped with nano electrospray ion source. Further details are reported in Supplementary Material and Methods.

### Validation of interactions by confocal microscopy

IGROV-1/Pt1 clone 14 KO transduced with USP18/UBP43 lentiviral particles were seeded in 12-well plates containing circular coverslips slides (15mm). Twenty-four h later, cells were fixed in 2% paraformaldehyde (Thermo Scientific) for 10 min at room temperature and then permeabilized in 0.1% Triton-X-100-PBS (Sigma-Aldrich) for 10 min. After washing in PBS and saturation in 2% BSA for 1 h, cells were incubated with the primary antibodies against USP18 (MyBiosource, San Diego, California, USA) and XPC (Invitrogen, Thermo Fisher) or RAD50 (Santa Cruz Biotechnology, Dallas, Texas, USA) diluted in 2% BSA-PBS overnight at +4 °C. At the end of incubation, samples were washed in PBS and then incubated with the secondary antibodies conjugated with Alexa 488 (for USP18) and Alexa 594 (for XPC or RAD50) for 1 h at room temperature, counterstained with Hoechst 33342 for 2 min and finally mounted with Prolong Gold AntiFade Reagent (Thermo Fisher). Samples were left to dry overnight at room temperature and then images were collected using a confocal laser scanning microscope Leica TCS SP8 X (Leica Microsystems GmbH, Mannheim, Germany). The data were analyzed using the software Leica LAS AF rel. 3.3 (Leica Microsystems GmbH) and the protein co-localization was measured by means of the Pearson correlation coefficient.

### Computational design of USP18-targeting peptides

Molecular modeling was performed using the USP18-ISG15 complex structure (PDB ID: 5CHV) as a template 33. Key binding interfaces were visually identified and extracted using Chimera X. Peptide candidates were generated by isolating ISG15 segments forming the most stable contacts with USP18 and constructing 3D models using the MAESTRO (Schrödinger Suite) Build module. Chimeric sequences were obtained by linking two binding motifs: TAT-conjugated variants, peptides 002, 004, 006, were assembled via a GAG linker to ensure membrane permeability. Control peptides were created by substituting basic residues with Alanine within the binding motif. All designed sequences are reported in Supplementary Table S3.

### Surface plasmon resonance analysis

Surface plasmon resonance (SPR) was used to study the interaction of peptides with the purified human USP18 protein (Cusabio Technology, Houston, TX, USA). Analyses were performed using a Biacore T200 platform (Cytiva, Marlborough, MA, US). The antibody anti-HIS Tag was immobilized on a CM5 sensor chip (Cytiva) via standard EDC (N-ethyl-N-(3-dimethylaminopropyl) carbodiimide)/NHS (N-hydroxysuccinimide) coupling employing the Amino Coupling Kit (Cytiva) and following the manufacturer's protocol. One flow cell served as a reference, undergoing the same activation and blocking steps without protein immobilization. After activation of the chip surface with EDC/NHS, excess carboxyl groups were blocked with 1 M ethanolamine (pH 8.0). The flow rate for all assays was set at 30 µL/min, and the sensor surface was regenerated with 50 mM NaOH after each injection. After anti-HIS tag antibody immobilisation, the USP18 protein is loaded and captured by its HIS tag on the chip (around 650 RU). The inhibitory and scrambled peptides were then injected at 1 µM. To analyze binding responses, Biacore T200 Evaluation Software (Cytiva) was used, reporting results as response units (RU) over time.

### Statistical analyses

Statistical analysis of preclinical data was performed using GraphPad Prism version 10 (Boston, MA, USA) using One-Way ANOVA with Bonferroni's test for multiple comparison. As regards analysis of the human sample cohort, statistical analysis was performed considering USP18 expression relatively to RPS13 and GAPDH, in terms of log2RQ (i.e., -ΔCt). The non-parametric Kruskal-Wallis (KW) or Wilcoxon (W) tests were used to examine the association between the USP18 levels and the main clinic-pathological characteristics. COMET data were evaluated by computing the ratio between the cross-link indexes of the H_2_O_2_ treated samples and that of the cisplatin and H_2_O_2_ treated samples subtracted for basal damage (i.e., cross-link ratio). Differences in the cross-link ratio distributions were assessed by Kruskal-Wallis test, followed by Bonferroni comparison for contrast estimates. For *in vivo* studies, a two-way ANOVA including the interaction term between factors was performed on the log10 transformed tumor volumes at the last time point in which all statistical units were still present. Wilcoxon's non-parametric tests were used to explore specific differences among groups of animals, with p-values estimated through exact tests. Statistical analyses of *in vivo* and human studies were performed using SAS Studio Software (Release 5.2; SAS Institute, Inc., Cary, NC, USA).

## Results

### Identification of DUBs essential for the growth of platinum-resistant cells

**Figure 1:**
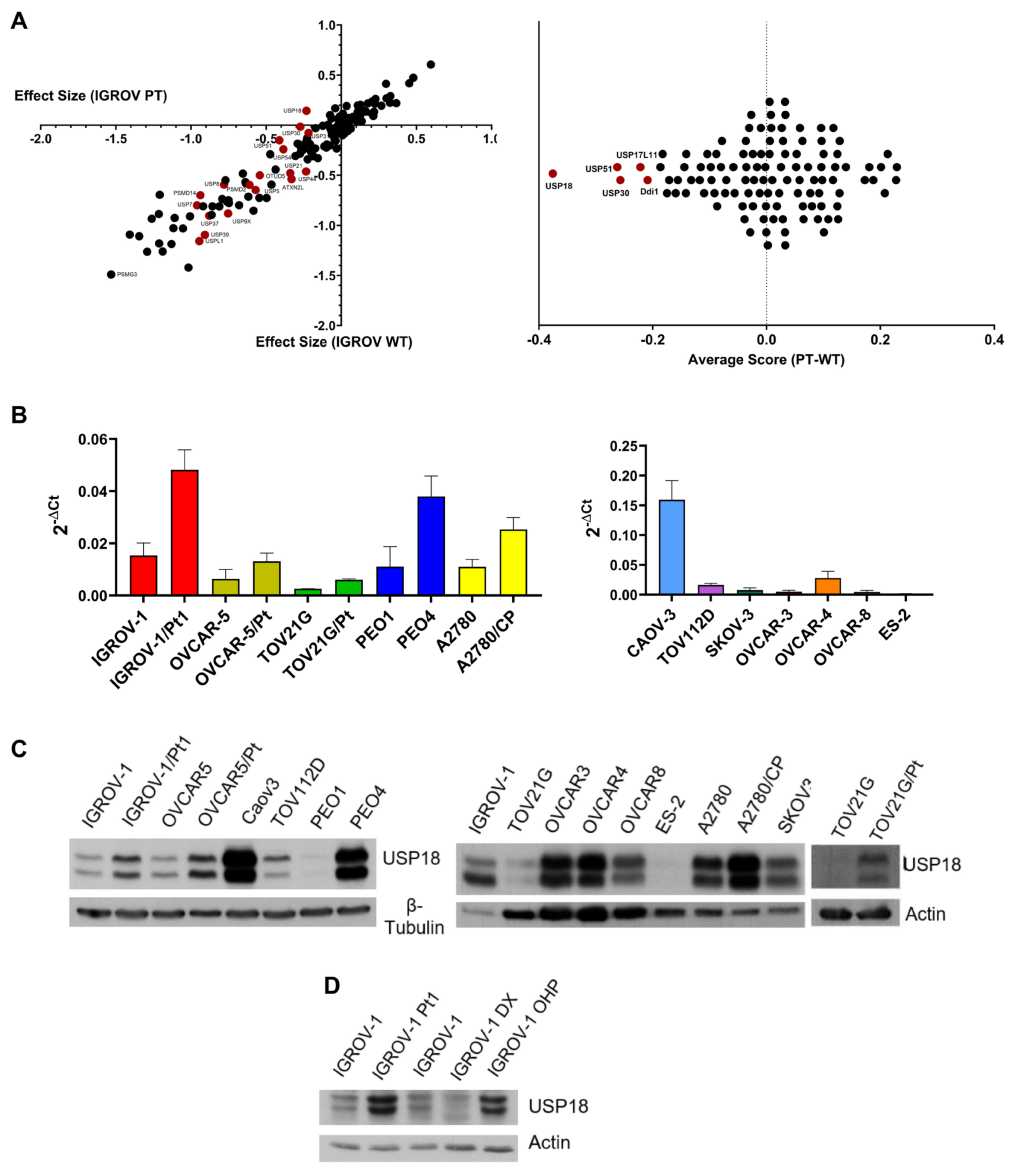
** Role of USP18 in cisplatin-resistant cells and expression in different ovarian carcinoma cell lines.** (**A**) Cas9-expressing IGROV-1 and IGROV-1/Pt1 cells were transduced with a custom library targeting DUBs. After 21 days, the transduced cells were expanded, harvested, and sequenced. Effect sizes are based on the guide representation at 5 days and 21 days post-transduction, with differences observed between IGROV-1/Pt1 and IGROV-1 cells across two independent experiments. The left panel shows the effect sizes from the two independent experiments. Effect size corresponds to the change in guide representation from the early (5 days) to the late (21 days) sample for each target gene. The average change in guide abundance per gene is plotted with IGROV-1/Pt1 on the x-axis and IGROV-1 on the y-axis. A negative effect size indicates guide depletion and essentiality. The right panel displays the average score of IGROV-1/Pt1 (PT) versus IGROV-1 (WT) cells. (**B**) Quantitative RT-PCR analysis of USP18 mRNA expression in a large panel of ovarian cancer cell lines, normalized to RPS13 housekeeping gene. Mean ± SD of 6 independent experiments are reported in the histograms. p-value< 0.001, Student's t test of parental versus resistant cells. (**C-D**) Western blot analysis of USP18 protein levels in different ovarian carcinoma cell lines and their resistant variants. Actin or β-tubulin were used as loading control. The quantification of band intensity is reported in Supplementary Figure S3.

To identify DUBs selectively required for the survival of cisplatin-resistant ovarian carcinoma cells, we performed a CRISPR/Cas9 dropout screen in the cisplatin-sensitive IGROV-1 cell line and its resistant derivative, IGROV-1/Pt1. Both cell lines were transduced with lentiviral constructs encoding Cas9, followed by a custom pooled sgRNA library targeting 87 characterized DUBs. Comparative analysis revealed that, among all DUBs screened, USP18 exhibited a marked dependency in the cisplatin-resistant IGROV-1/Pt1 cells, with minimal impact in the parental IGROV-1 cells. Specifically, the average dropout score for USP18 was -0.23 in IGROV-1/Pt1 cells compared to 0.14 in IGROV-1 cells, indicating a resistance-specific vulnerability (Figure 1A).

Other DUBs with higher dropout in the resistant cells included PSMD14/RPN11/POH1 - a component of 19S regulatory proteasome cap complex that mediates substrate deubiquitination with average scores of -0.94 in IGROV-1/Pt1 and -0.70 in IGROV-1, suggesting a stronger dependency in the resistant context. In contrast, several DUBs, such as USP7, USP39, USP37, and USP10, scored highly in both cell lines, indicating a general dependency on these genes irrespective of cisplatin resistance. When we used a similar approach in the cisplatin-sensitive PEO1 and cisplatin-resistant PEO4 cells, comparative analyses revealed that USP1 exhibited a marked dependency in resistant cells (Supplementary Figure S2). However, we elected to focus on USP18-related mechanisms because USP1 role in drug resistance was previously reported 14.

### Elevated USP18 expression is observed in various platinum-resistant cells and is associated with poor outcome

To gain insights into the possible role of USP18 in cisplatin resistance, we examined its mRNA levels by qRT-PCR in a panel of ovarian cancer cell lines, including pairs of cisplatin-sensitive and -resistant cells. We found that the levels of this DUB were enhanced in the IGROV-1/Pt1 variant as well as in additional cisplatin-resistant sublines including the OVCAR5/Pt, the PEO4, the A2780/CP and the TOV21G/Pt as compared to the corresponding parental cell lines (Figure 1B). Increased mRNA levels paralleled protein levels as shown by western blot analysis. The augmentation of the USP18 protein involved the two forms migrating as 34 and 39 kDa protein known to represent the N-terminal truncated (USP18-sf) and full-length isoforms (Figure 1C; Supplementary Figure S3) 34. The enhancement of USP18 expression was specific for platinum-resistant cells as it was evident in oxaliplatin-resistant IGROV-1/OHP cells, but not in doxorubicin-resistant IGROV-1/DX cells (Figure 1D; Supplementary Figure S3). An analysis of the TCGA database further supported a role for USP18 in ovarian carcinoma biology 18. Indeed, survival (progression free-survival) of patients with tumors that display high USP18 expression was lower than that of patients with tumors showing low expression both when considering all histological subtypes and endometrioid cases (Supplementary Figure S4A and B**)**, respectively. We also examined mRNA levels in clinical ovarian carcinoma specimens from a case material containing around 60% cases of high-grade serous ovarian carcinoma and around 20% of endometrioid carcinomas (Supplementary Table S2). The relative expression levels were associated with tumor grade (KW p-value: 0.041), but not with other parameters such as diagnosis (KW p-value: 0.485) and tumor stage (KW p-value: 0.320) (Supplementary Figure S5).

### Loss of function studies support a role for USP18 in platinum-drug resistance

**Figure 2:**
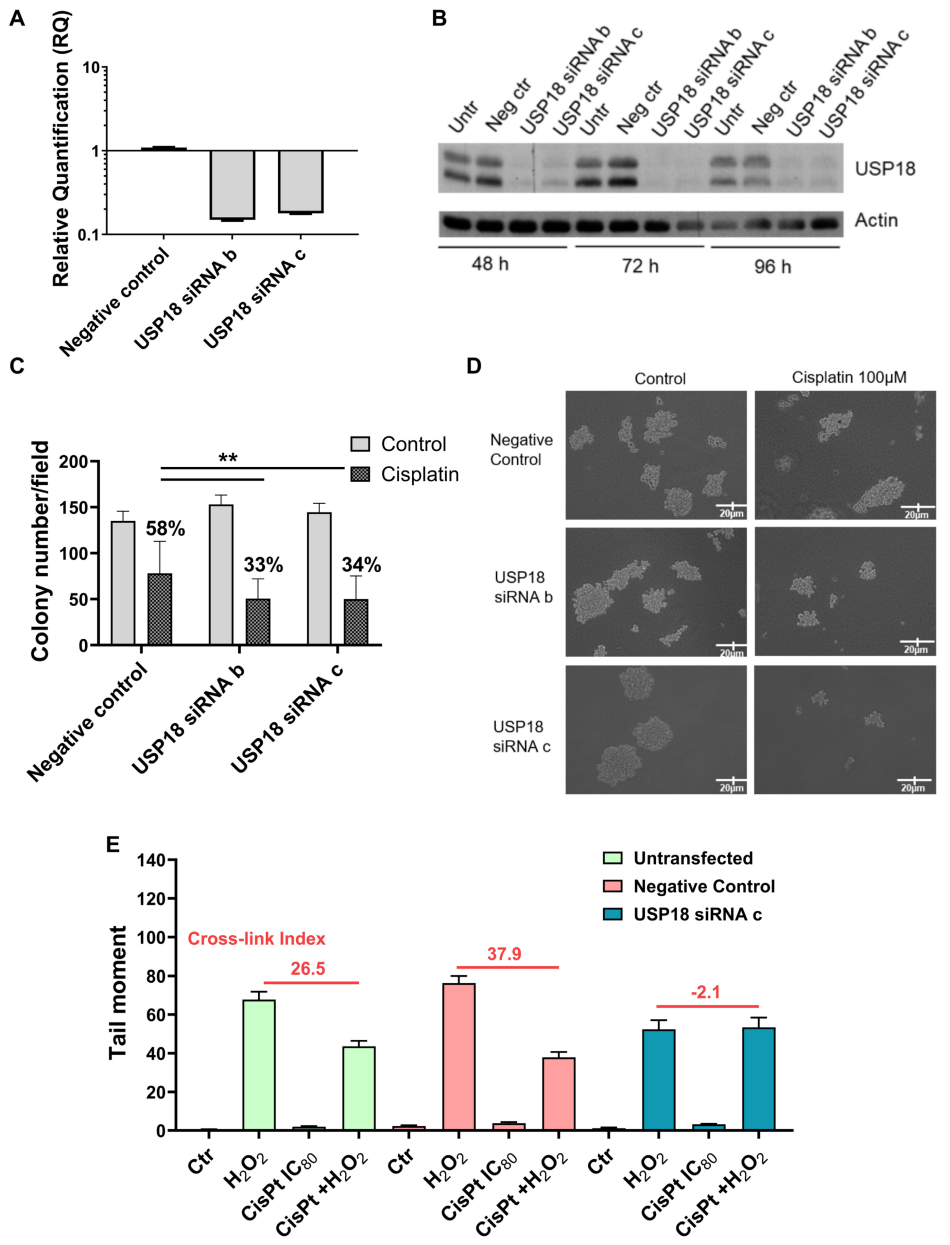
** USP18 loss of function studies in IGROV-1/Pt1 cells.** IGROV-1/Pt1 cells were transiently transfected with two different siRNAs directed against different regions of USP18 (siRNA b and siRNA c). (**A**) USP18 mRNA levels were analyzed 48 h after transfection start by qRT-PCR; untransfected cells were used as calibrator; GAPDH was the housekeeping gene. (**B**) Western blot analysis of USP18 protein levels at different times after siRNAs transfection. Actin was used as loading control. Forty-eight hours after transfection start cells were treated with cisplatin for 1 h and then (**C**) seeded for colony forming ability assay in soft-agar and incubated for almost 2 weeks; percent of cell survival of treated cells versus control cells are reported above the histograms (mean ± SD of technical replicates). The statistical analysis was performed by One-way ANOVA with Bonferroni's correction test. ** p-value < 0.01 negative control cells* versus* USP18 siRNA b and siRNA c cells treated with cisplatin, (**D**) representative images of colonies in soft-agar; EVOS microscopy, magnitude 10x; or (**E**) 24 h after treatment end harvested for modified COMET assay to measure cross-links for quantitative analysis of DNA damage; breaks are induced by H_2_O_2_ added to the agarose; in the histogram is reported a tail moment (mean ± SEM).

**Figure 3:**
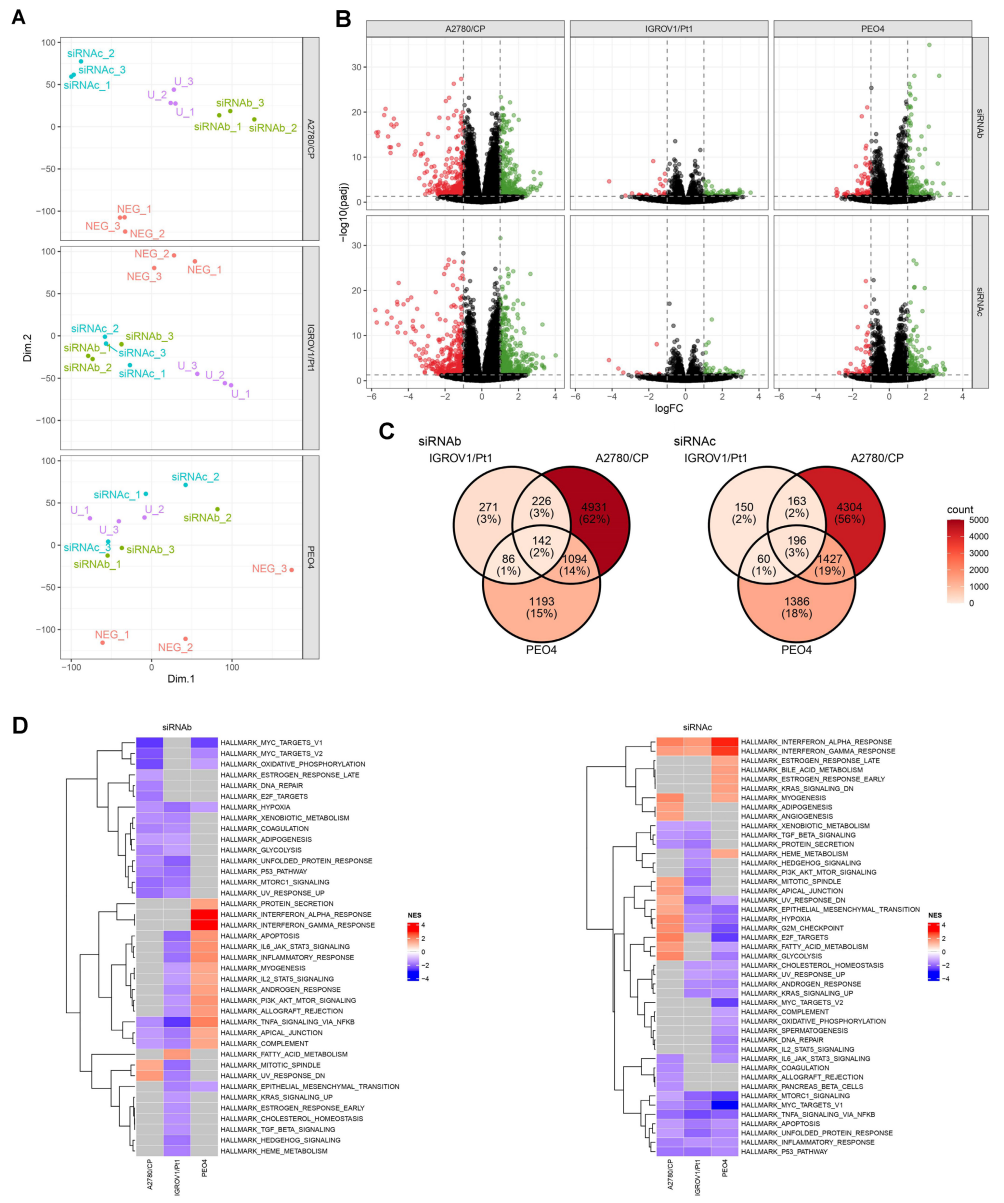
** RNA-seq analysis in ovarian cancer resistant cells after USP18 knockdown.** RNA-seq analysis in IGROV-1/Pt1, A2780/CP and PEO4 cells was performed 48 h after USP18 transfection. (**A**) Principal component analysis for each cell line. Each sample is colored according to its condition. (U = untransfected cells). (**B**) Volcano plot [x axis : logFC; y axis : -log10(padj)]. Each column of the panel corresponds to a cell line, and each row to a siRNA (b or c). Each panel shows the results of the differential gene expression analysis between the negative control and the corresponding siRNA in the corresponding cell line. Cut-off for the colors (logFC = 1; padj = 0.05). (**C**) Venn diagram showing differentially expressed genes in common between each cell line, depending on the USP18 knockdown. (**D**) Heatmaps of the normalized enrichment score of a GSEA for each siRNAs on the Hallmark gene set collection. Red boxes indicates a positive enrichment, blue boxes a negative enrichment and grey boxes a non significant enrichment.

Given that USP18 role in survival of platinum- resistant cells was identified in the IGROV-1/Pt1 cell line, we used this as a model for loss of function studies. At first, we carried out knockdown of USP18 by transfection with 3 siRNAs (siRNA a, siRNA b and siRNA c). Subsequent experiments were carried out with the most efficient ones (siRNA b and c) and were designed to examine the phenotypes of the silenced cells as compared to untransfected and negative siRNA transfected cells (Figure 2). We observed a marked downregulation of USP18 mRNA 48 h post-transfection (Figure 2A), as well as a decrease of USP18 protein levels as evident in time course western blot experiments (Figure 2B). We found that USP18 silencing resulted in increased sensitivity to cisplatin, as shown by clonogenic assays in soft agar (Figure 2C-D). USP18 silenced cells also displayed impaired ability to repair DNA damage as detected through a modified COMET assay adapted to quantitatively measure cisplatin-induced DNA cross-links because DNA breaks artificially induced by adding hydrogen peroxide to the samples 20 are reduced by cisplatin cross-linking ability. Thus, decreased cross-linking ability suggests the presence of higher levels of DNA strand breaks. Indeed, cisplatin cross-linking ability was impaired upon USP18 silencing because the cross-link index decreased indicating higher levels of damage in silenced cells (mean cross link index: -2.1) than in untransfected (mean cross link index: 26.5) and negative control transfected siRNA cells (mean cross link index: 37.9) (Figure 2E). This different cross-linking ability was confirmed when evaluated in terms of cross link ratio (KW p-value: < 0.001), with statistically higher values (contrast W p-value: < 0.001) in the negative control treated samples. To examine the pathways related to USP18 in resistant cells, we performed RNA-seq analysis in various drug-resistant cell lines upon USP18 knockdown (Figure 3). We compared the transcriptomic profiles from negative control-siRNA and USP18 siRNAs-transfected cells. Principal component analyses clearly identified the groups, indicating the effects induced by silencing (Figure 3A). The differential gene expression analysis revealed that 142 and 196 genes constituted a set of modulated genes common to IGROV-1/Pt1, A2780/CP and PEO4 cells by siRNAb and siRNAc, respectively (Figure 3B and 3C). Heatmap of gene expression fold change for the modulated gene set shared by the three cell lines shows that the genes were mostly regulated in the same way, regardless of the cell line or siRNA used (Supplementary Figure S6). Gene set enrichment analysis (GSEA) revealed a statistically significant positive enrichment for Interferon alpha and Interferon gamma response gene sets in IGROV-1/Pt1, A2780/CP and PEO4 cells with siRNAc. However, these changes in gene expression were significant only in PEO4 cells with siRNAb (Figure 3D). In addition, the analysis highlighted the negative enrichment for the gene sets of several pathways related to apoptosis process (in all three cell lines with siRNAc, in IGROV-1/Pt1 cells with siRNAb) and DNA repair (in A2780/CP with siRNAb, in PEO4 cells with siRNAc).

### Phenotypic characterization of USP18 knockout clones generated by CRISPR/Cas9 approach

**Figure 4:**
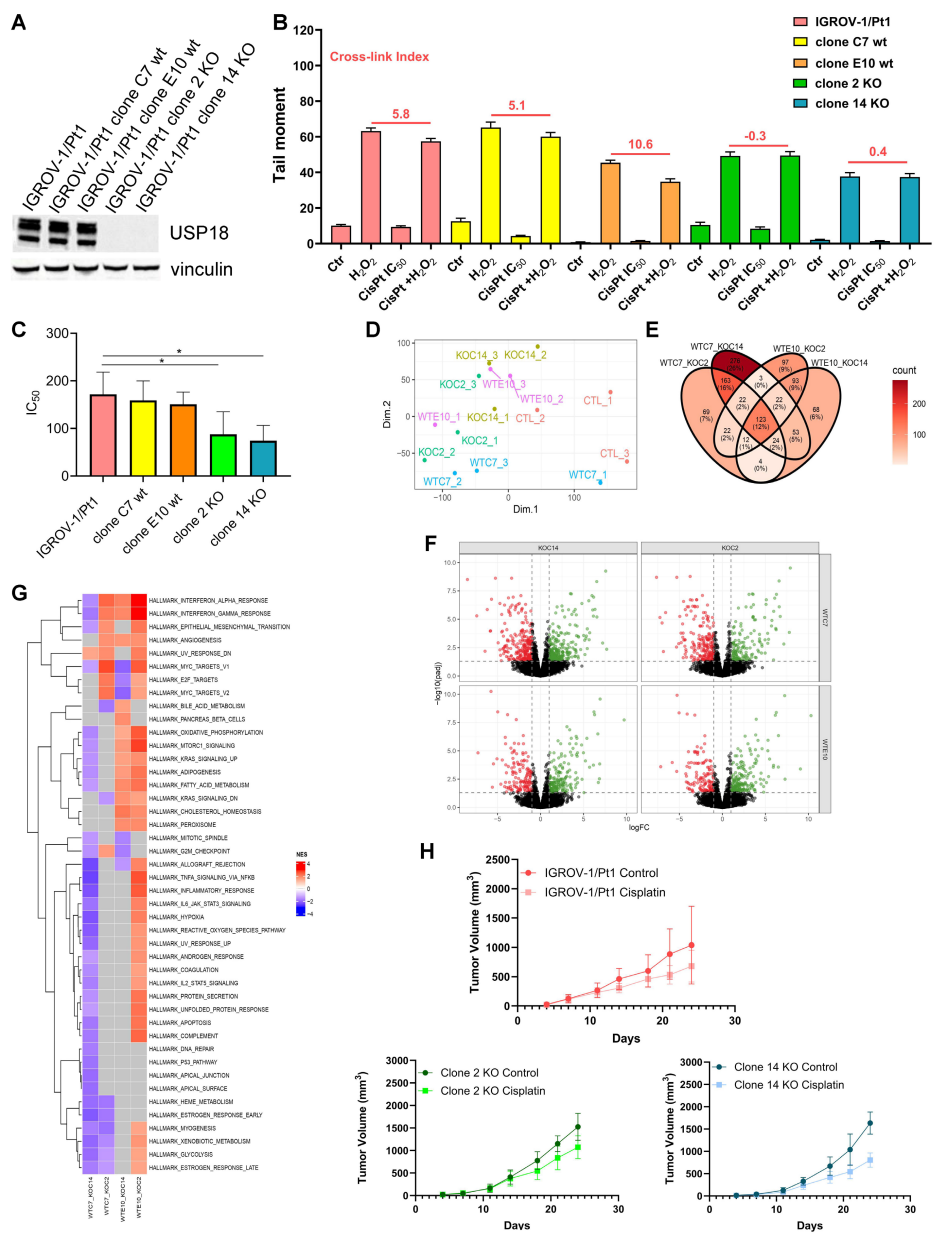
** Phenotypic characterization of CRISPR/Cas9 USP18 knockout clones.** (**A**) Western blot analysis of USP18 protein expression in IGROV-1/Pt1 cells and in different clones (wt and USP18 KO) generated by CRISPR/Cas9 approach starting from this cell line. Vinculin was used as loading control. Phenotypic studies of CRISPR/Cas9 clones in term of DNA damage repair efficiency by modified COMET assay (**B**) and colony forming ability in soft-agar (**C**) after cisplatin treatment for 1 h and 24 h drug free; p-value < 0.05 by One Way ANOVA with Bonferroni correction. RNA-seq analysis in USP18 KO clones *versus* wt clones. (**D**) Principal component analysis for each cell line. Each sample is colored according to its condition (**E**) Venn diagram showing differentially expressed genes in common between each comparison. (**F**) Volcano plot [x axis : logFC; y axis : -log10(padj)]. Each column of the panel corresponds to a KO clone, and each row to a wt clone. Each panel shows the results of the differential gene expression analysis between the corresponding wt clone and the corresponding KO clone. (**G**) Heatmaps of the normalized enrichment score of a GSEA on the Hallmark gene set collection. Red boxes indicate a positive enrichment, blue boxes a negative enrichment and grey boxes a non significant enrichment. (**H**) Antitumor activity of cisplatin in athymic mice bearing human IGROV-1/Pt1 cells and IGROV-1/Pt1-derived USP18 KO clones. Animals bearing tumors were treated by i.p route with 4.5 mg/kg cisplatin every week for 3 weeks.

To clarify the contribution of USP18 to platinum drug resistance, we used a CRISPR/Cas9 approach to generate stable USP18 KO in the IGROV-1/Pt1 cell line as this was the cell line where USP18 essentiality was defined. Western blot analysis showed similar USP18 protein levels between IGROV-1/Pt1 and two control clones, C7 and E10, generated with control sgRNAs from the bulk IGROV-1/Pt1 population and designated as wild-type (wt), while USP18 protein was not expressed in the two KO clones (clone 2 and clone 14) (Figure 4A). Phenotypic studies indicated that USP18 KO clones have a higher sensitivity to cisplatin (Figure 4B) and increased DNA damage (mean cross-link index -0.3 and 0.4 for KO clone 2 and 14, respectively) compared to the parental IGROV-1/Pt1 cell line and wt clones as shown by decreased cross-link index (mean cross-link index 5.8 for IGROV-1/Pt1 and 5.1 and 10.6 for wt clone C7 and E10, respectively; Figure 4C). This different cross-linking ability was confirmed when evaluated in terms of cross link ratio (KW p-value: <0.001), with statistically higher values (contrast W p-value: < 0.001) in IGROV-1/Pt1 and wt clones versus KO clones. Rescue of USP18 expression in clone 14 indicated the loss of sensitization (IC_50_ = 143.9 ± 2.3 µM). Principal component analysis demonstrated no clear group clustering, highlighting the common origin of the clones from a single bulk population (Figure 4D). Heatmap of gene expression fold change of the modulated genes shows that these genes are mostly regulated in the same way across comparisons, except for the genes HOXD11, CNKSR1, PCSK5, CGREF1, PPP1R14C and TMC5 that were downregulated when the KO clones were compared to the wt C7 clone and up-regulated when the KO clones were compared to the wt E10 clone, highlighting clonal specific features. RNA-seq analysis in KO clones indicated differences for the GSEA depending on the wt clone (Figure 4E-F). We still found a positive enrichment for genes from the Interferon alpha and Interferon gamma response gene sets (except for clone C7 wt vs clone 14 KO), and a negative enrichment for gene sets related to DNA repair and apoptosis only for the comparison clone C7 wt vs clone 14 KO (Figure 4G; Supplementary Figure S7).

The antitumor efficacy of cisplatin was tested in athymic mice bearing s.c. the human ovarian carcinoma IGROV-1/Pt1 as well as USP18 KO clones (clone 2 and clone 14) and control clones (clone C7 wt and clone E10 wt). The treatment started 5 days after cell injection, when tumors were established. No antitumor effects were achieved by cisplatin in mice bearing IGROV-1/Pt1 as well as clone C7 and clone E10 (Figure 4H and Supplementary Figure S8). Conversely, at day 21, the USP18 KO clone 14 tended to respond to cisplatin with a TVI of 48%. By testing for the simple main effects of treatment for groups, a significant effect (p-value = 0.049) was observed for the Clone 14 (Figure 4H). This finding was confirmed also by applying Wilcoxon's non-parametric test (p-value = 0.017) that revealed also a borderline effect for Clone 2 (p-value = 0.105). Cisplatin treatment was well tolerated as shown by mice weight over time (Supplementary Figure S8).

### Proteomics analysis of USP18 interactors

To clarify possible mechanisms leading to USP18-mediated resistance, we explored USP18 interacting proteins using a proteomic analysis with affinity purifications followed by mass spectrometry. We immunopurified Flag-tagged USP18 (DDK-USP18) from whole-cell extracts prepared from the USP18 KO clone 14 engineered to express USP18-DDK tagged by a lentiviral vector. As a negative control, the same clone was infected with an empty vector. Immunoprecipitations were performed in three biological replicate experiments, and associated proteins were digested on beads and identified by liquid chromatography combined with mass spectrometry. Table 1 shows the interactors identified. Among them, 2 proteins involved in DNA repair were detected i.e., XPC and RAD50.

**Figure 5:**
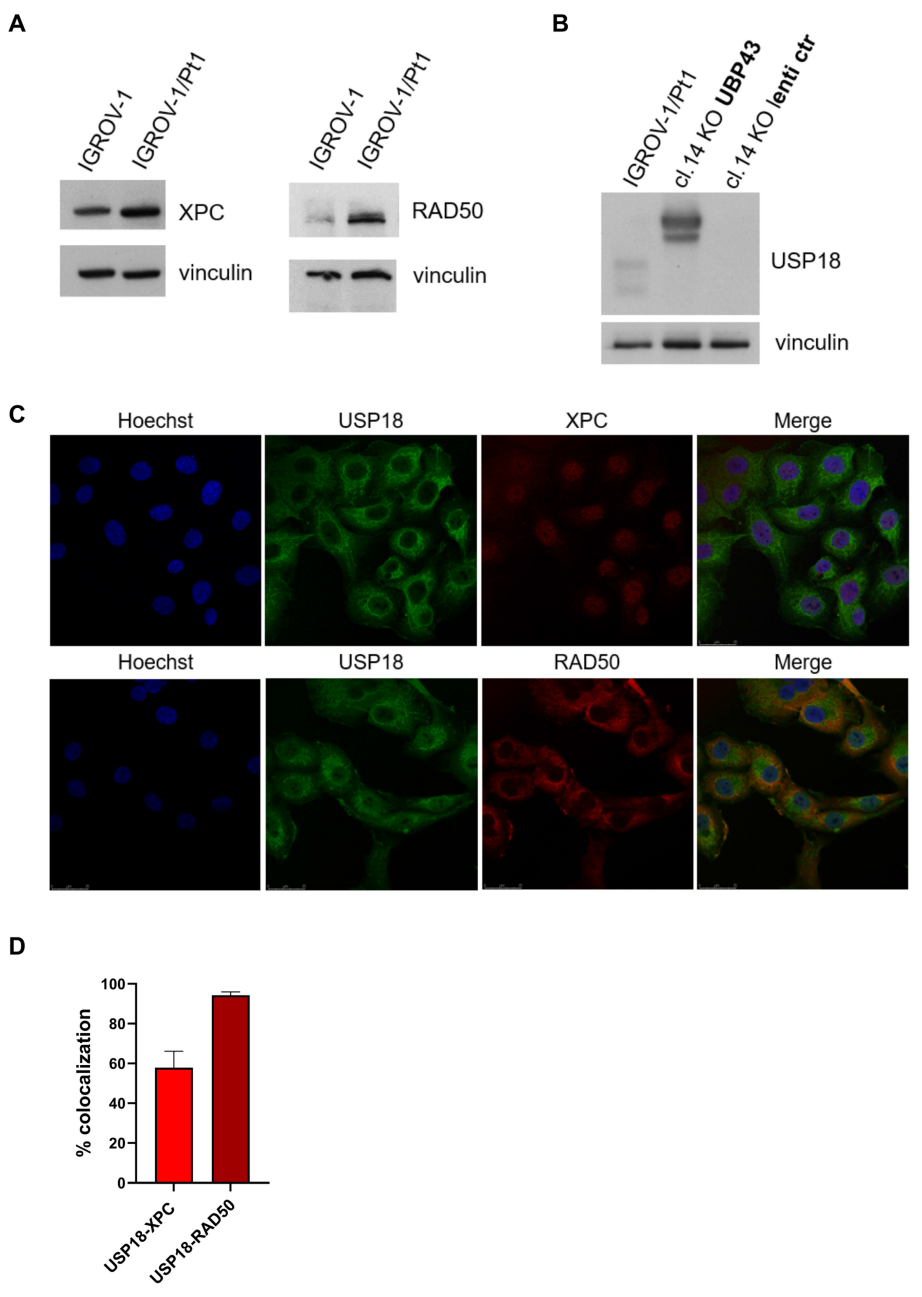
** Validation of USP18 interactors, XPC and RAD50, identified by proteomics analysis. (A)** Western blot analysis of XPC and RAD50 protein levels in IGROV-1 and its resistant variant IGROV-1/Pt1. Vinculin was used as a loading control (**B**) Western blot analysis of USP18 protein levels in the parental IGROV-1/Pt1 cells and this cell lines transduced with lentiviral particles with empty vector (lenti ctr) or UBP43 (USP18). Vinculin was used as loading control. (**C**) Representative confocal images of IGROV-1/Pt1 knock out clone 14 engineered to overexpress USP18/UBP43, cells with staining for USP18 (green), XPC or RAD50 (red), Hoechst (blue) and the merged image. (**D**) The histogram indicates co-localization between USP18 and XPC or RAD50 proteins (mean ± SD of at least 3 different fields).

The protein expression level of the two interactors, XPC and RAD50 was evaluated by western blot analysis. We found an increased expression in IGROV-1/Pt1 cells compared to the parental cell line IGROV-1 (Figure 5A). The cellular localization of the two proteins was examined by confocal microscopy. In IGROV-1/Pt1 clone 14 KO transduced with USP18/UBP43 (Figure 5B), the Pearson correlation for XPC was 0.47 ± 0.05 (Figure 5C), indicating that a colocalization of USP18 and XPC was approximately 60% (Figure 5D), compared to IGROV-1/Pt1 (co-localization around 45%; Supplementary Figure S9). In IGROV-1/Pt1 clone 14 KO transduced with USP18/UBP43 (Figure 5B), the Pearson correlation for RAD50 was 0.7 1 ± 0.03 (Figure 5C), indicating that a colocalization of USP18 and RAD50 was almost 95% (Figure 5D), compared to IGROV-1/Pt1 (co-localization around 89%; Supplementary Figure S9).

### USP18 targeting by peptides

**Figure 6:**
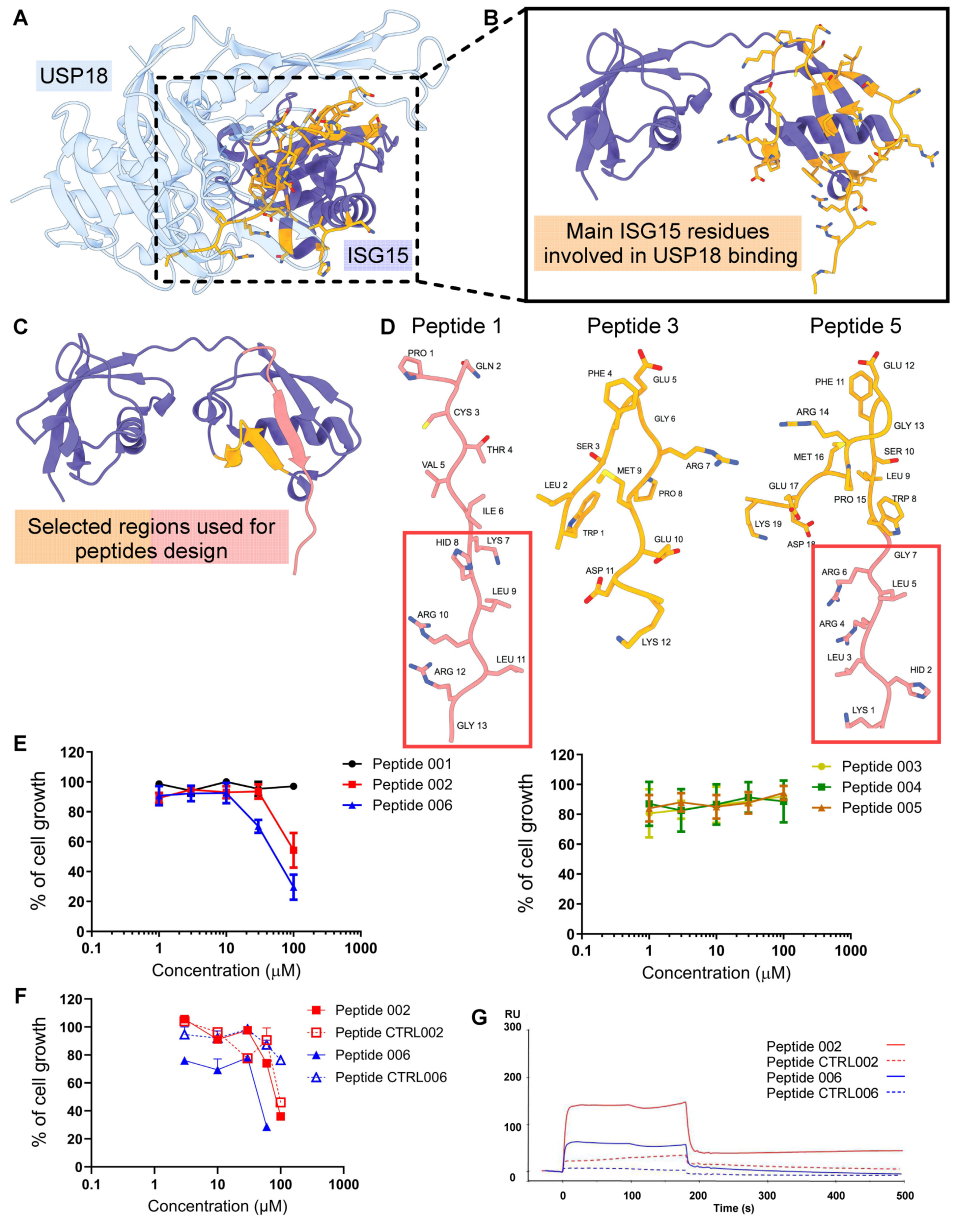
** Structural modelling, antiproliferative activity and target interaction of USP18-derived peptides.** (**A**) Crystal structure of the human USP18-ISG15 complex (USP18 in light blue, ISG15 in purple; interface residues of ISG15 shown in orange). (**B**) Close-up of the most extensive USP18-ISG15 contacts, highlighting key ISG15 residues (orange) interacting with USP18 (purple). (**C**) Two distinct ISG15 surface regions (red and orange) selected as templates for peptide design. (**D**) Modelled 3D structures of three representative inhibitory peptides, colored according to the region indicated in panel C. (**E-F**) IGROV-1/Pt1 cells were seeded and 24 h later exposed to USP18-derived peptides (or relative control) in serum-free medium for 24 h; at the end of incubation cells were washed in saline solution and added complete medium. Cell sensitivity was assayed 72 h after treatment start by cell counting. IC_50_ is the concentration inhibiting cell growth by 50%; Peptides 001-003-004-005 IC_50_ > 100µM; Peptide 002 IC_50_ = 100µM; Peptide 006 IC_50_ = 54.6µM (mean ±SD of 3 independent experiments). (**G**) Biacore sensorgram of the interaction between ligand USP18 coated sensorchip and the peptides used as analytes. In red the peptide 002 in blue the peptide 006; the dotted lines are the relative scrambled peptides. The graph shows the association phase (0-200 seconds) and the dissociation phase (200-400 seconds). Response units (RU) are reported on the Y-axis, while time (seconds) is reported on the X-axis.

Since we showed that USP18 is upregulated in cisplatin-resistant ovarian cancer cell lines, we investigated the possibility to target this enzyme by rationally design of peptide inhibitors. We started from the deposited co-crystal structure of murine USP18 bound to ISG15 (PDB 5CHV) 33 and inspected it to identify the key interacting residues. We found two ISG15 segments (amino acid 121-132 and 142-154) establishing multiple contacts with the catalytic core of USP18 (Figure 6A-C). We isolated these regions to obtain ISG15 peptide mimics able to bind USP18 and interfere with its enzymatic activity. We obtained two peptides 001 (aa 142-154) and 003 (121-132) from the identified ISG15-interacting regions and another peptide (peptide 005) by combining the sequence of peptide 001 with peptide 003 (Figure 6D). To ensure cellular uptake, we fused each compound, via a GAG linker, to the HIV-1 Tat protein cell penetrating peptide obtaining 002, 004 and 006 peptides (Supplementary Table S3). We initially carried out cell sensitivity assays with the designed USP18 inhibitors in the IGROV-1/Pt1 cell line. Cells were exposed to USP18 peptides for 24 h in serum-free medium and counted 72 h after treatment start. Peptides 002 and 006, sharing the KHLRLRG motif critical for interaction within USP18 catalytic groove and bearing the Tat peptide, demonstrated cell growth inhibitory activity at concentrations of ~100 µM and 54.6 µM, respectively (Figure 6E-F). Peptides lacking Tat sequence (001, 003 and 005) or KHLRLRG motif (004) showed no antiproliferative activity (Figure 6E-F). Control peptides (CTRL002 and CTRL006), designed to confirm sequence specificity, were generated by substituting key basic residues with Alanine, thereby impairing electrostatic interactions. Treatment of cisplatin-resistant IGROV-1/Pt1 cells with the latter indicated lack of antiproliferative activity for CTRL006, where, at the highest concentration tested, CTRL002 reduced cell growth suggesting a potential role also for the PQCTVI motif. When the antiproliferative activity of the peptides was tested in additional cell lines including pair of cisplatin-sensitive and -resistant cells (A2780 and A2780/CP, OVCAR5 and OVCAR5/Pt), we observed a behavior similar to what observed in IGROV-1/Pt1 cells, as only peptides 002 and 006 displayed growth inhibitory activity (Supplementary Figure S10, Supplementary Table S4). To characterize the interaction of peptides with the target protein, we elected to use SPR analyses. The obtained results demonstrate a specific interaction between USP18 and the two peptides, as observed through the Biacore sensorgrams; the initial rising phase of the curve corresponds to the association rate constant (ka), which reflects how quickly the analyte binds to the immobilized ligand. In our case, this association was very rapid, indicating a strong initial affinity. The subsequent decline in the curve represents the dissociation rate constant (kd), which measures how quickly the analyte detaches from the ligand. A rapid dissociation was observed, suggesting that although binding occurs quickly, the analyte does not remain bound for an extended period, indicating a reversable transient interaction. No binding was evident with scrambled peptides (Figure 6G).

## Discussion

In the present study, we used a CRISPR/Cas9 DUB dropout screen to identify which DUBs are critical in supporting the survival of cisplatin-resistant ovarian carcinoma cells. With this approach, we found that several DUBs are required for survival of resistant cells (i.e., USP8, USP18, USP30, USP51, USP1). The biological relevance of specific DUBs seems to be in part dependent on the experimental model/histological subtype as evidenced by the comparison of the results obtained with the endometroid carcinoma IGROV-1 and IGROV-1/Pt1 cell lines with those from the high-grade serous PEO1 and PEO4 cells. In the latter model systems, we observed an association of USP1 with survival of PEO4 resistant cells. This is in keeping with reports from the literature supporting USP1 contribution to drug resistance 14. Several USPs have been implicated in cisplatin resistance through the modulation of DNA damage repair, apoptosis, and cell survival signaling, including USP1, USP7, USP8, USP10, and USP14. These enzymes together with components of the ubiquitin proteasome system like PSMC6 19 enhance cellular tolerance to cisplatin-induced DNA damage and promote survival under genotoxic stress 13. Overall, the mechanisms appear to be peculiar for each DUB in keeping with the preference of these enzymes for specific substrates. For instance, USP1 acts on the FANCD2/PCNA complex, supporting tolerance to cisplatin-induced DNA damage 35, while USP7 promotes cisplatin resistance by facilitating DNA damage repair through deubiquitination of key DNA damage response factors 35. Regarding endometroid carcinoma cell systems, we previously reported that USP8 is key in sustaining cisplatin resistance and that its contribution to tumor cell survival, occurring by stabilization of HER family receptors and apoptosis-related proteins, can be counteracted by both molecular and pharmacological tools 15, 36. USP10 stabilizes HDAC6 and confers cisplatin resistance in non-small cell lung cancer, whereas loss of USP10 enhances cisplatin-induced apoptosis 37. The proteasome associated USP14 DUB has been shown to confer platinum resistance by stabilizing the transcription factor Bcl-6 38 as well as by impacting on connexin 32 39. USP14 has also been reported to confer cisplatin resistance in ovarian cancer, making it a promising druggable target, as USP14 inhibition restores cisplatin sensitivity 40. Similarly, functional studies have shown that PSMC6 knockdown reduces cell growth and clonogenic potential, particularly in cisplatin-resistant ovarian cancer cells, and increases sensitivity to cisplatin 19. These effects are linked to the accumulation of ubiquitinated proteins and reduced ERK1/2 signaling via upregulation of DUSP6. Therefore, PSMC6 has been proposed as a novel proteasome-related therapeutic target 19. These findings highlight both shared and unique roles of USP family members in shaping cisplatin resistance and underscore USP18 as a mechanistically distinct contributor. Therefore, in the present study, based on the score indicating dependency on DUBs for survival, we focused our attention on USP18. Compared with other USPs, USP18 primarily regulates ISGylation and interferon-associated signaling pathways 41. This protein is well characterized for its role in the antiviral response 42, whereas its potential involvement in drug resistance is poorly understood. Some evidence implicates USP18 in the response to radiation 43, implying that it may modulate DNA repair mechanisms activated following the treatment with platinum compounds.

To dissect the role of USP18 in cisplatin resistance, we first examined its expression at the mRNA and protein level in a panel of ovarian carcinoma cell lines of different histotypes and found that its levels were consistently enhanced in 5 cisplatin-resistant variants, including the IGROV-1/Pt1 cells where the CRISPR-Cas9 dropout screen was carried out. Of note, the association between resistance and USP18 seems to be specific for platinum agents as no enhancement was observed in doxorubicin-resistant cells, whereas a mild increase was evident in IGROV-1/OHP cells, selected for resistance to oxaliplatin 17. When evaluating USP18 in clinical specimens, we found that it was associated with tumor grade, implying a role in aggressiveness of tumor cells.

When performing phenotypic analyses in IGROV-1/Pt1 cells using loss of function approaches, we observed a sensitization to the effect of cisplatin by colony forming assays in knockdown cells and KO clones in association with reduced cross-linking ability of cisplatin. The cross-linking ability of cisplatin can be measured by using a modified COMET assay where DNA breaks are artificially induced by adding hydrogen peroxide to the samples before processing them 20. Decreased cross-linking ability of cisplatin suggests that higher levels of DNA strand-breaks are present. Here, higher levels of DNA breaks observed in cisplatin-treated silenced or KO cells may be responsible for sensitization to the drug. Of note, antitumor activity studies using cisplatin in the platinum-resistant IGROV-1/Pt1 model indicated enhanced drug efficacy when USP18 KO clones were xenografted in nude mice.

A transcriptomic analysis in knockdown IGROV-1/Pt1 cells in addition to that in 2 other cisplatin-resistant variants, i.e., A2780/CP and PEO4 cells, indicated that USP18 controls a variety of pathways in cisplatin-resistant cells. Some pathways are common across different cellular models and somehow expected based on the available knowledge on USP18. Indeed, the most well characterized role of this DUB is the inhibition of the IFN response 42, so that USP18 is a main regulator of innate immune response. Such observations were corroborated by RNA-seq analysis in USP18 KO clones as compared to wt clones derived from the bulk IGROV-1/Pt1 population. In keeping with our findings, a recent report in triple negative breast cancer suggests that inhibition of USP18 induces interferon and cytokine response as well as MHC1 antigen presentation, thereby implying an enhancement of cancer immunogenicity 44.

To dissect mechanistic aspects of USP18 role in drug resistance, we carried out a proteomic analysis in IGROV-1/Pt1 USP18 KO cells engineered to express USP18/UBP43 by lentiviral vector infection. Such an analysis revealed that USP18 interacts with various cellular proteins including proteins involved in the repair of cisplatin-induced damage such as XPC and RAD50. XPC, a key gene in the early step of DNA repair by NER, has been shown to be up-regulated by Tie-1 in ovarian cancer in association with acquisition of cisplatin resistance 45. RAD50 acts in multiple steps of homologous recombination including DNA end resection and RAD51 loading 46. RAD50 targeting has been reported to sensitize breast cancer cells to cisplatin 47, and its deficiency has been proposed as a predictor of platinum sensitivity in sporadic epithelial ovarian cancers 48. Besides, a role for USP18 in DNA repair has been shown in relation to radiation-induced damage 43. In fact, hypersensitivity to radiation was observed in USP18 KO colon carcinoma cells. Such a behavior was hypothesized to be related to inactivation of Adenosine Deaminase RNA Specific function. Of note, in this context USP18 disruption resulted in enhanced sensitivity to type I IFN.

A promising observation of the present study was that treatment of the IGROV-1/Pt1 cisplatin-resistant cells with ISG15-derived peptides directed to USP18 reduced cell growth. In our assays, we observed a better performance for peptide 6 whose scramble sequence resulted inactive, in keeping with the binding profile determined with SPR analyses; peptide 2 was less effective and the corresponding scramble sequence was able to produce cell growth inhibition consistently with possible off-target effects. Such effects were evident also in other ovarian carcinoma cell lines. In all tested cellular models, additional peptides lacking the Tat sequence or KHLRLRG motif did not inhibit cell proliferation. Further effort is required to improve the current peptide design to enhance binding stability through optimization of key contact residues and modulation of structural rigidity, thereby improving target selectivity.

Our results support the hypothesis that USP18 contributes to chemoresistance and can be targeted by short, rationally designed peptides. The development of peptides is an emerging approach that could be extremely effective in molecular therapy 49.

**Figure 7:**
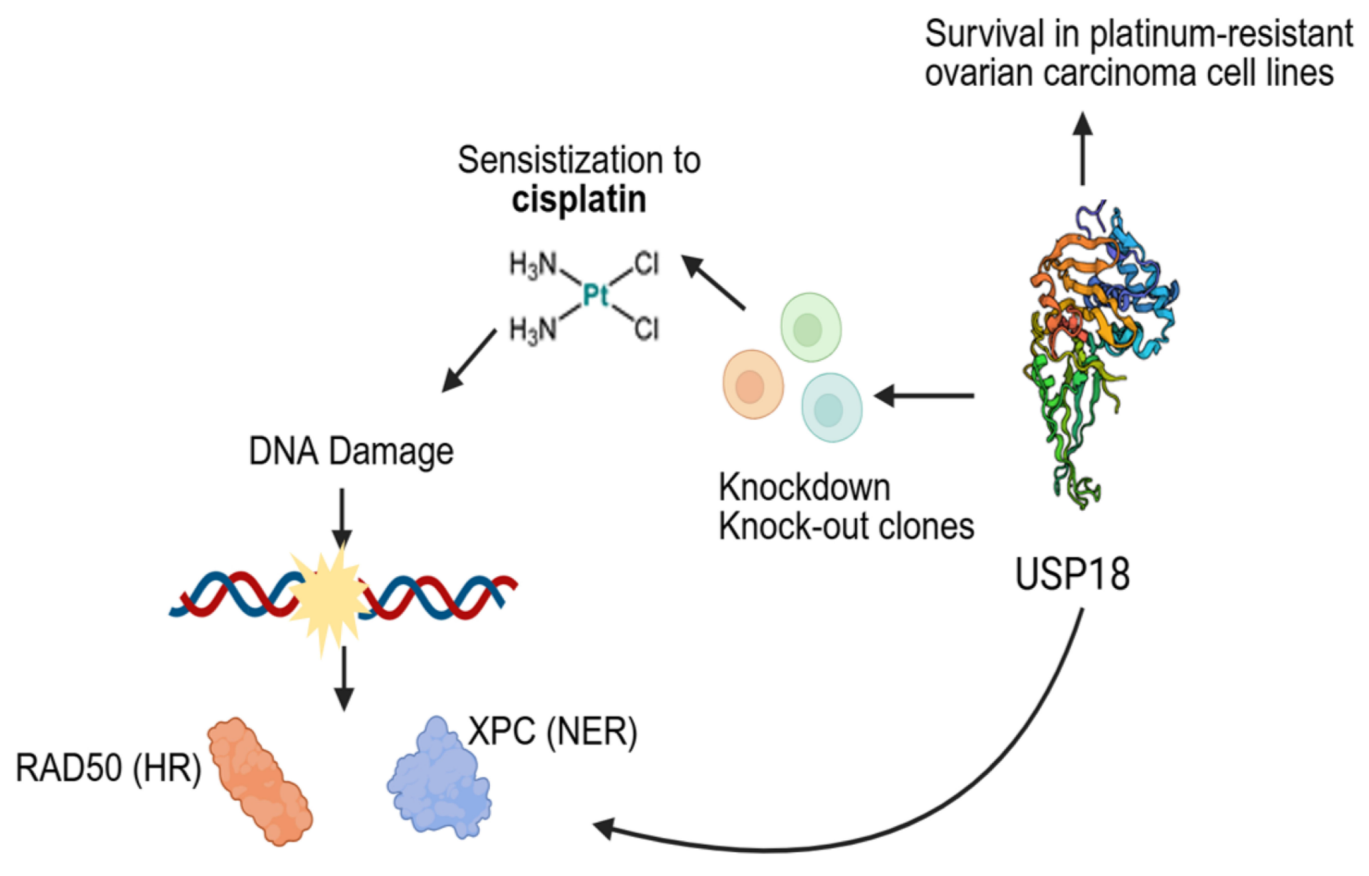
** Proposed model of USP18 role in survival of platinum-resistance ovarian carcinoma cell lines.** USP18 is a critical mediator of survival in cisplatin-resistant ovarian carcinoma cells. USP18 knockdown or knockout induce sensitization to cisplatin in association with reduced cross-linking ability. Proteomic analysis revealed two important USP18 interactors, XPC and RAD50, involved in DNA repair mechanisms. Created with Biorender.com.

In conclusion, here we show that USP18 is required for survival of cisplatin-resistant ovarian carcinoma cells (Figure 7). Given that USP18 KO clones tended to be more sensitive to cisplatin than wt clones both *in vitro* and *in vivo*, USP18 may represent a biomarker useful to predict the efficacy of platinum-based therapy. USP18 biological role appears to be mediated by the interaction with DNA repair proteins. Targeting USP18 by specific peptides may allow to design new therapeutic approaches against platinum-resistant ovarian cancer.

## Supplementary Material

Supplementary figures and tables.

## Figures and Tables

**Table 1:** Proteomic analysis of IGROV-1/Pt1 clone 14 KO transduced with USP18/UBP43 cells versus empty-vector cells.

Biological Function	Protein
RNA regulation, transcription and translational, protein synthesis	BolA-like protein 2 Isoform B of DnaJ homolog subfamily B member 6 La-related protein 1 Nuclear cap-binding protein subunit 1 Nucleolar complex protein 4 homolog Nucleolar protein 7 [OS=Homo Sapiens] Origin recognition complex subunit 3 Protein polybromo-1 Ras GTP-ase-activating-like protein IQGAP3 Replication factor C subunit RNA-binding protein 8A Signal peptidase complex subunit 2 SNW domain-containing protein 1 SURP and G-patch domain-containing protein 2 U3 small nucleolar RNA-associated protein 14 homolog A WD repeat-containing protein 74
DNA Damage Repair, Cell Death	DNA repair protein complementing XP-C cells DNA repair protein RAD50 Metaxin-2
Calcium Signaling	45 kDa calcium-binding protein Isoform HMG-Y of High mobility group protein HMG-I/HMG-Y Reticulocabin-2
Epigenetics	Glutamate -rich WD repeat-containing protein 1 Histone H2A type 2-B Histone H3.3
Microtubule Dynamics Regulation	Microtubule cross-linking factor 1 Serine/Threonine-protein kinase MARK2

All samples have been analyzed using Dionex Ultimate 3000 nano-LC system (Sunnyvale CA, USA) connected to an Orbitrap Exploris™ 240 or to an Orbitrap Fusion™ Tribrid™ Mass Spectrometer (Thermo Scientific, Bremen, Germany) equipped with nano electrospray ion source. Protein abundance ratio (USP18-DDK versus empty) is equal to 100 for all protein shown [OS=Homo sapiens]. Top hits are shown.

## Data Availability

The data discussed in this publication have been deposited in NCBI's Gene Expression Omnibus (Corno et al.; 2026) and are accessible through GEO Series accession number GSE326132 (https://www.ncbi.nlm.nih.gov/geo/query/acc.cgi?acc=GSE326132).

## Abbreviations

PARP: poly-ADP-ribose polymerase
VRAC: volume-regulated anion channel
BRCA1/2: BReast CAncer
DUBs: deubiquitinases
USP: ubiquitin-specific peptidase
ISG15: interferon-stimulated gene 15
DCTD: Division of Cancer Treatment and Diagnosis
FBS: fetal bovine serum
BFP: blue fluorescent protein
TCGA: The Cancer Genome Atlas
sgRNA: single guide RNA
KO: knockout
wt: wild-type
OTM: olive tail moment
GSEA: gene set enrichment analysis
TVI: tumor volume inhibition
PBS: phosphate buffered saline
BSA: bovine serum albumin
GAG: group-specific antigen
SPR: surface plasmon resonance
rt-PCR: real time-polymerase chain reaction
KW: Kruskal-Wallis
W: Wilcoxon
XPC: Xeroderma Pigmentosum C
RAD50: double-strand breaks repair protein 50
ka: constant association
kd: constant dissociation
IFN: interferon
MHC1: major histocompatibility complex class 1
NER: nucleotide excision repair
