# Supplementary material for: USP18 Impacts Cisplatin Resistance in Ovarian Cancer Cells by Modulating DNA Repair
Source: Int J Biol Sci. 2026 May 29;22(11):5780–98. doi: 10.7150/ijbs.130399 (PMC13282739; doi:10.7150/ijbs.130399)
Supplement: Supplementary file 1 — Supplementary figures and tables. [file ijbsv22p5780s1.pdf]

Supplementary Material

USP18 impacts on cisplatin resistance in ovarian cancer cells by modulating DNA repair

Cristina Corno[1], Matteo Costantino[1], Pietro Pettinari[1], Luca Mirra[1], Simone Stucchi[1], Noemi Arrighetti[1], Nunzio Perta[2], Giovanni Di Muccio[2], Mathilde Robin[3], Giovanni L. Beretta[1], Elisabetta Corna[1], Nives Carenini[1], Loredana Cleris[1], Diego Colombo[4], Elena Luison[5], Chiara Maura Ciniselli[6], Mara Lecchi[6], Paolo Verderio[6], Mariangela Figini[5], Stig Linder[7,8], Diego Tosi[3,9], Anna La Teana[2], Padraig D'Arcy[7] and Paola Perego[1*]

[1] Molecular Pharmacology Unit, Department of Experimental Oncology, Fondazione IRCCS Istituto Nazionale dei Tumori, via Amadeo 42, 20133 Milan, Italy

[2] Polytechnic University of Marche,Department of Life and Environmental Science, via Brecce Bianche, 60131 Ancona,Italy

[3] Early Clinical Trial Unit, Medical Oncology Department, Institut régional du Cancer Montpellier, Inserm U1194, Montpellier University, France

[4] Department of Medical Biotechnology and Translational Medicine, University of Milan, via Saldini 50, 20133 Milan, Italy

[5] Department of Advanced Diagnostics, Fondazione IRCCS Istituto Nazionale dei Tumori, via Amadeo 42, 20133 Milano, Italy

[6] Unit of Bioinformatics and Biostatistics, Department of Epidemiology and Data Science, Fondazione IRCCS Istituto Nazionale dei Tumori, via Venezian 1, 20133 Milan, Italy

[7] Department of Biomedical and Clinical Sciences (BKV)
SE-581 83, Linköping

[8] Division of Biochemistry, Department of Medical Biochemistry and Biophysics, Karolinska Institutet, Stockholm, Sweden

[9] Fondazione Gianni Bonadonna, via Bertani 14, 20154 Milan, Italy

*Corresponding author: paola.perego@istitutotumori.mi.it

Supplementary Material and Methods

Proteomic analysis

Cells were plated in two 20 cm diameter plates. After 2 days (70% confluency), they were washed 3 times with PBS-Vanadate (0.1 mM) and 1mL of lysis buffer (Hepes 10 mM, EGTA 0.1 mM, EDTA 0.1 mM, $MgCl_2$ 2 mM, KCl 10 mM, NP40 1.5%, DDT 1 mM and halt protease inhibitor cocktail 1X) was added to the cells. Lysis was performed for 30 min on ice. Protein lysates were transferred in a 15 ml tube, incubated on ice for 45 min and mixed by vortex every 10 min. Each sample was passed 5 times in 21 gauge syringe and sonicated for 30 sec. After, protein lysates were centrifuged at 10000 g for 10 min at 4°C. Supernatants were taken and salt concentration was adjusted to resemble PLC Buffer (Hepes 50 mM pH 7.9, NaCl 150 mM, Glycerol 10%, EGTA 1 mM). Protein concentration was evaluated using the BCA assay (ThermoFisher). Meanwhile, 200 µL of Sepharose Protein A hydrated in TNT Buffer (Tris-HCl 20 mM pH 7.4, NaCl 150 mM, TritonX-100 1%) was centrifuged, washed with TNT buffer and incubated with protein lysates and primary Antibody (Anti –DDK flag 1:200 of lysate's volume). Sepharose protein A, lysates and Ab mixed were kept in rotation at 4°C overnight. Then, samples were centrifuged at 1000 g for 1 min at 4°C and washed 3 times with 500 µL HTNG Buffer (Hepes 20 mM pH 7.9, NaCl 150 mM, glycerol 10%, Triton X-100 0.1%, halt protease inhibitor cocktail 1X) and 3 times with HTNG Buffer without glycerol. Triton X-100 was removed by washing 3 times with Hepes 20 mM pH 7.9, NaCl 150 mM. Samples were frozen at -80°C until proteomic analysis. Parallel samples were eluted to check for the IP efficiency. Elution was performed adding Laemli Buffer (Tris-HCl pH 6.8 260 µL, SDS 10% 500 µL, H_2O 240 µL). Samples were boiled for 5 min and centrifuged at 15000 g for 3 min. Supernatants were taken and used for SDS electrophoresis.

Forty µg of proteins in 50 mM ammonium bicarbonate (50 µL total volume) were reduced using 2.5 µL of 100 mM dithiothreitol (5 mM final concentration) at 55 °C for 30 min, and then alkylated with 5 µL of 150 mM iodoacetamide (15 mM final concentration) at room tempetrature for 20 min in the dark, prior to digestion with 6 µL of trypsin 0.1 µg/µL in 50 mM ammonium bicarbonate at 37 °C overnight with final reaction blocking in 50% formic acid (1 µL). The protein lysate (10 µL/sample) was then purified (desalted) with a 5 µg zip-tip C18 column and the eluted material was concentrated in a speedvac and reconstituted in 20 µL with 0.1% formic acid for nLC-HRMS analysis. Peptide mixtures were pre-concentrated onto an Acclaim PepMap 100™ column (100 µm x 2 cm C18 - Thermo Scientific) and separated on EASY- Spray column ES902A, 25 cm x 75 µm ID packed with Thermo Scientific Acclaim PepMap RSLC C18, 3 µm, 100 Å using mobile phase A (0.1 % formic acid in water) and mobile phase B (0.1% formic acid in acetonitrile 20/80, v/v) at a flow rate of 0.300 µL/min.

The elution program (min, % B) was: 0.0, 4.0%; 3.0, 4.0%; 103.0, 28%; 113.0, 40%; 114.0, 95%; 117.0, 95%, 120.0, 4%; 123.0, 4%; 126.0, 95%; 129.0, 95%; 132.0, 4%; 135.0, 4%; 138.0, 95%; 141.0, 95%; 144.0, 4%; 150.0, 4%. The temperature was set to 35°C and the samples were injected in triplicates into the Orbitrap Fusion spectrometer. The sample injection volume was 6 µL. Two blanks were run between samples to prevent sample carryover. MS spectra were collected over an m/z range of 375 – 1500 Da at 120,000 resolution, operating in the data dependent acquisition mode. HCD was performed with collision energy set at 35 eV. Polarity: positive. All mass spectrometry data were analyzed with Proteome Discoverer 2.5 software (Thermo Fisher) using UniProt protein sequence database with Homo sapiens taxonomy (sp_incl_isoforms TaxID = 9606_and_subtaxonomies; v2024-09-13) and with enzyme digestion specificity set to trypsin. For the analysis the following settings were used. Dynamic modifications: max equal modifications per peptide = 3; max dynamic modifications per peptide = 4; dynamic modification = oxidation (+15.995 Da, M). Static modifications: carbamidomethyl (+57.021 Da, C). Dynamic modifications (N-terminal modifications): acetyl (+42.011 Da, N-terminus); met-loss+acetyl (-89.030 Da, M); met-loss (-131.040 Da, M). For label-free quantification (LFQ) the following filter were used: (protein level) peptides ≥ 2; (peptide level) xcorr ≥ 2.2, rank=1, confidence=high; (peptide-spectrum matches level, PSMs) xcorr ≥ 2.2. The general quantification settings used were: peptides to use = Unique + Razor. Quantitative rollup and hypothesis testing were: a) protein abundance calculation = summed abundances; b) protein ratio calculation = pairwise ratio based; c) imputation mode = none; d) hypothesis test = t-test (background based). The obtained data are reported in Results section and Supporting Information. The found proteins are described by their uniprot code (accession number), name (description), percentage of the amino acids of the found peptides with respect to those of the entire protein (Coverage %), number of different peptides for a protein group (Peptides), number of identified peptides (Peptide Spectrum Matches - PSMs), number of peptides unique to a protein group (Unique peptides), peptides shared between different groups (Razor peptides) and other calculated parameters (AAs, MW, pI).

MTS assay

Different ovarian carcinoma cell lines, IGROV-1, OVCAR5 and A2780, and corresponding -resistant variants (IGROV-1/Pt1, OVCAR5/Pt, A2780/CP) were tested for cell sensitivity to USP18-derived peptides by MTS assays; cells were seeded in 96-well plates and then treated for 24 h with different concentrations of peptides in serum-free medium. Fresh completed medium was then added for 48 h. Seventy-two hours after treatment start, the MTS reagent (Promega) was added to medium and incubated for 3 h. Absorbance was measured at 490 nm with plate reader. IC_{50} values represent the drug concentrations producing 50% cell growth decrease. USP18-derived peptides (Covalab) was diluted in distilled water.

Table S1. Basic information about the primary and secondary antibodies used in the manuscript.

**Antibody Name**	**Target antigen**	**Supplier/ catalog #**	**Host species**	**Clonality**
Anti-Actin antibody	Actin	Sigma<br># A2066	rabbit	monoclonal
Anti-vinculin antibody (clone hVIN-1)	Vinculin	Sigma<br>#V9131	mouse	monoclonal
Anti-beta Tubulin antibody-Loading Control	Beta-Tubulin	Abcam<br>#ab6046	rabbit	polyclonal
Anti-USP18 (D4E7) mAb	USP18	Cell Signaling<br># 4813	rabbit	monoclonal
Anti-RAD50 (G-2)	RAD50	Santa Cruz<br># sc-74460	mouse	monoclonal
Anti-XPC (3.26) antibody	XPC	Invitrogen<br># MA1-23328	mouse	monoclonal
Amersham<br>ECL anti-mouse IgG<br>ECL anti-rabbit IgG	**Secondary Ab peroxidized**	GE Healthcare<br># NA931<br># NA934	 sheep<br>donkey	

Table S2. **Clinical and pathological characteristics of ovarian cancer patients enrolled in the pilot study** as described previously (Costantino et al., Int J Biol Sci 2025)

	N	%
**Diagnosis**		
**Endometrioid carcinoma**	25	18.66
**High-grade serous carcinoma**	80	59.70
**Other[a]**	29	21.64
**Grade**		
**1**	15	11.19
**2**	13	9.70
**3**	106	79.10
**Stage**		
**I**	26	19.40
**II**	21	15.67
**III**	71	52.99
**IV**	16	11.94

[a] Other, Mucinous adenocarcinoma, Clear cell carcinoma, Low-grade serous carcinoma

IGROV-1/Pt 1

GCTCTTTGGCATCAGAACGGATTACATGAATACACATTCCTCCTTCTCTAATTCTTGCCT 60

ACCCGCATTGTATTTTCACAGAGATTCCATCGTGCCTGGCTCACATAAGCGCTTCCTGGA 120

AGTGAAGTCGTGCTGTCCTGAACGCGGGCCAGGCAGCTGCGGCCTGGGGGTTTTGGAGTG 180

ATCACGAATGAGCAAGGCGTTTGGGCTCCTGAGGCAAATCTGTCAGTCCATCCTGGCTGA 240

GTCCTCGCAGTCCCCGGCAGATCTTGAAGAAAAGAAGGAAGAAGACAGCAACATGAAGAG 300

AGAGCAGCCCAGAGAGCGTCCCAGGGCCTGGGACTACCCTCATGGTCATTAGACCCCTCC 360

CGTTTTCCTCTTCTTGACTGCATGTAAATGTTCGGCTCACCCCCTCCGCTCTGAAGCCGC 420

AGAGCTTTGTATTCGACGGCTCAT**GCTTTCCTGTGCCTGAGTTT** 464

479 bp fragment

GCTCTTTGGCATCAGAACGGATTACATGAATACACATTCCTCCTTCTCTAATTCTTACCT 60

ACCCGCATTGTATTTTCACAGAGATTCCATCGTGCCTGGCTCACATAAGCGCTTCCTGGA 120

AGTGAAGTCGTGCTGTCCTGAACGCGGGCCAGGCAGCTGCGGCCTGGGGGTTTTGGAGTG 180

ATCACGAATGAGCAAGGCGTTTGGGCTCCTGAGGCAAATCTGTCTGGGCTCCTGAGGCAA 240

ATCTGTCTGGGCTGCTCTCTCTTCATGTTGCTGTCTTCTTCCTTCTTTTCTTCAAGATCT 300

GCCGGGGACTGCGAGGACTCAGCCAGGAGAGCGTCCCAGGGCCTGGGACTACCCTCATGG 360

TCATTAGACCCCTCCCGTTTTCCTCTTCTTGACTGCATGTAAATGTTCGGCTCACCCCCT 420

CCGCTCTGAAGCCGCAGAGCTTTGTATTCGACGGCTCAT**GCTTTCCTGTGCCTGAGTTT** 479

381 bp fragment

GCTCTTTGGCATCAGAACGGATTACATGAATACACATTCCTCCTTCTCTAATTCTTGCCT 60

ACCCGCATTGTATTTTCACAGAGATTCCATCGTGCCTGGCTCACATAAGCGCTTCCTGGA 120

AGTGAAGTCGTGCTGTCCTGAACGCGGGCCAGGCAGCTGCGGCCTGGGGGTTTTGGAGTG 180

ATCACGAATGAGCAAGGCGTTTGGGCTCCTGAGGCAAATCTGTCAGTCCAGAGCGTCCCA 240

GGGCCTGGGACTACCCTCATGGTCATTAGACCCCTCCCGTTTTCCTCTTCTTGACTGCAT 300

GTAAATGTTCGGCTCACCCCCTCCGCTCTGAAGCCGCAGAGCTTTGTATTCGACGGCTCA 360

T**GCTTTCCTGTGCCTGAGTTT** 381

Supplementary Figure S1. Exon 1 DNA sequence of IGROV-1/Pt1 and USP18 KO clones. Exponentially growing cells were harvested and genomic DNA was extracted. After PCR amplification of the genomic region encompassing the CRISPR-Cas9 editing site, PCR products were purified from an agarose gel and sequenced. Yellow: exon 1 DNA sequence. The start codon (ATG) is indicated in red; Green: CRISPR/Cas9 guide annealing sequence; Blue: DNA sequence inserted by the CRISPR/Cas9; Bold: primer sequences used for PCR amplification and sequencing.

Supplementary Table S3. A detailed list of the amino acid sequences of the generated peptides.

Peptide Identifier	Peptide Sequence
**001**	PQCTVIKHLRLRG
**002**	GRKKRRQRRRPQ-GAG-PQCTVIKHLRLRG
**CTRL002**	GRKKRRQRRRPQ-GAG-PQCTVIAGLALAG
**003**	WLSFEGRPMEDK
**004**	WLSFEGRPMEDK-GAG-GRKKRRQRRRPQ
**005**	KHLRLRGWLSFEGRPMEDK
**006**	KHLRLRGWLSFEGRPMEDK-GAG-GRKKRRQRRRPQ
**CTRL006**	AGLALAGWLSFEGRPMEDK-GAG-GRKKRRQRRRPQ

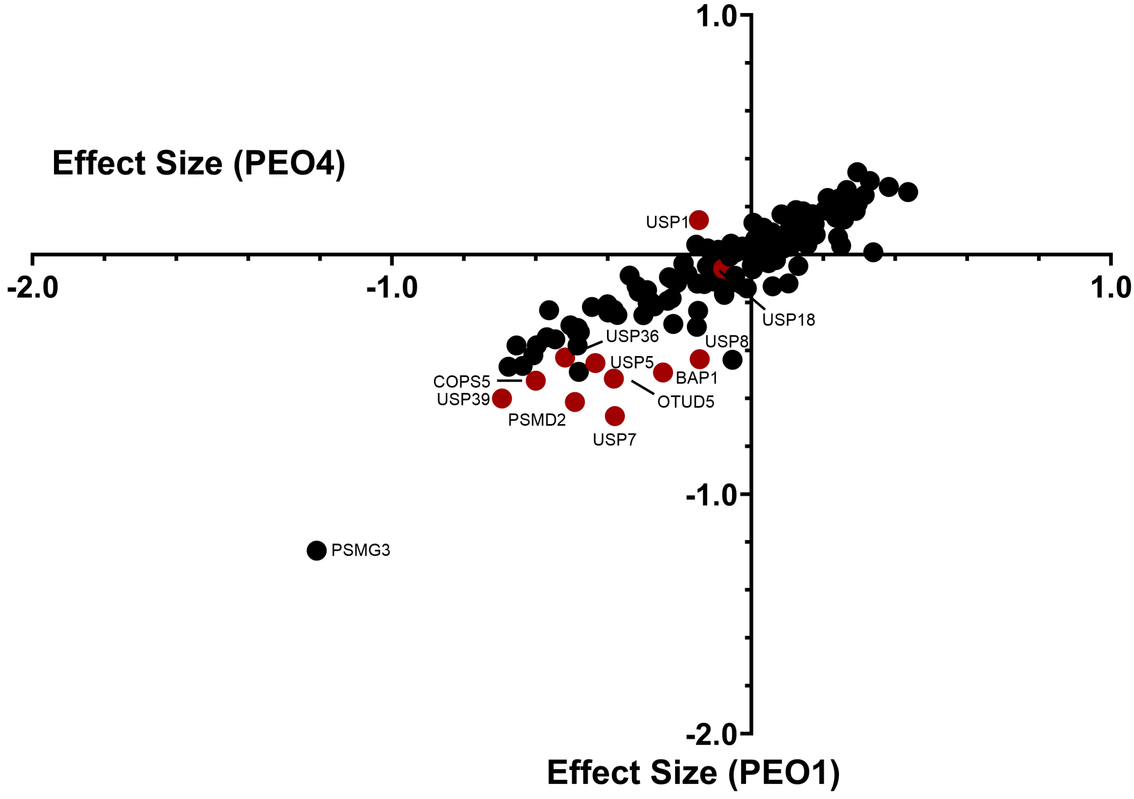

Supplementary Figure S2. CRISPR/CAS9 DUB KO screen in PEO1 vs PEO4. Cas9-expressing PEO1 and PEO4 cells were transduced with a DUB-targeting gRNA library. Cells were harvested at day 5 and day 21, and guide representation was quantified by sequencing. The average change in guide abundance per gene is plotted with PEO4 on the x-axis and PEO1 on the y-axis.

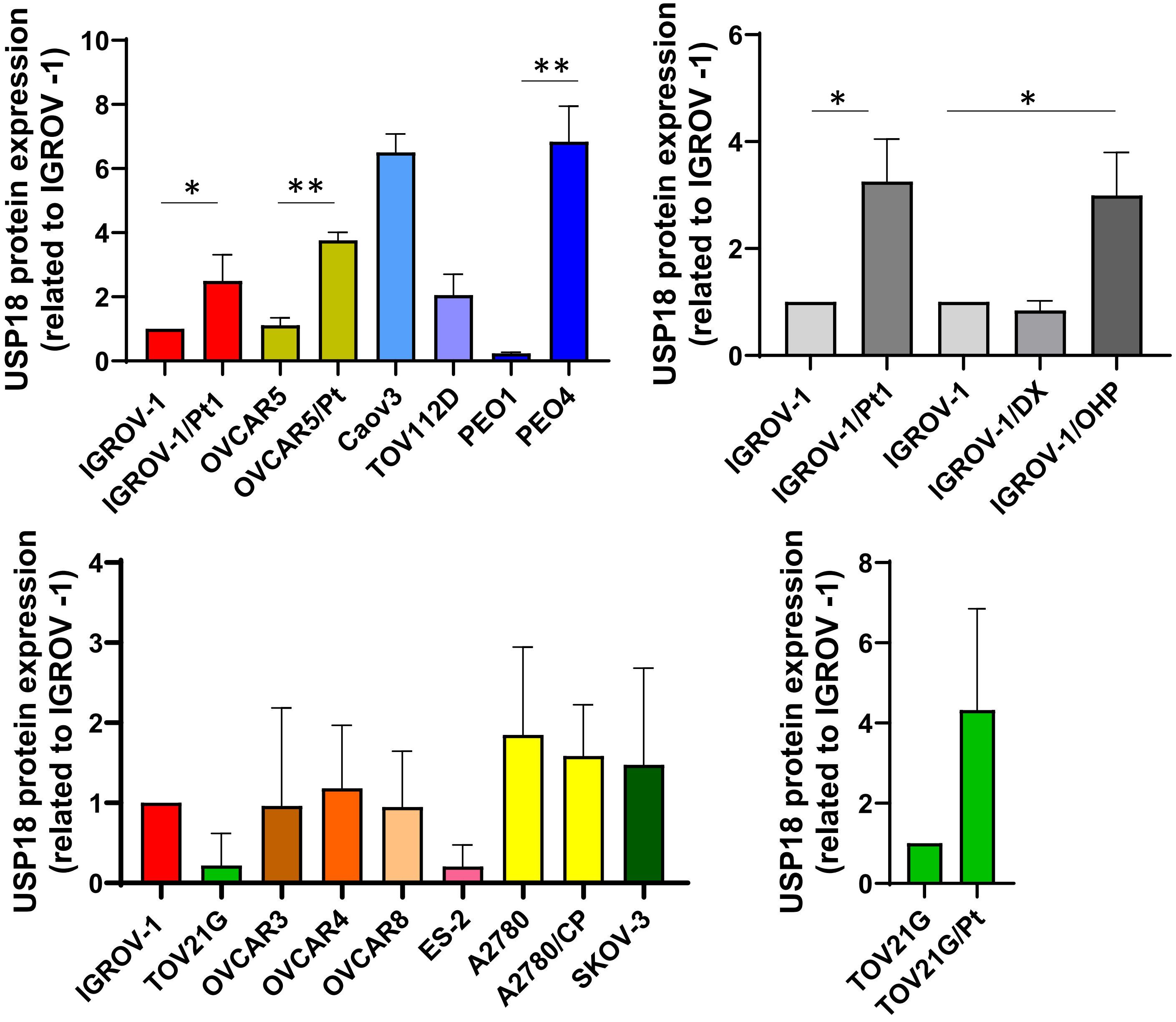

Supplementary Figure S3. Quantification of western blot analysis reported in Figure 1. USP18 protein expression in different ovarian carcinoma cell lines with respect to IGROV-1 cells (set to 1) is reported. The sum of the 2 bands was considered for USP18. Band intensity was quantified using ImageJ and normalized to β-tubulin or actin. Unpaired Student's t test results are as follows: IGROV-1 vs IGROV-1/Pt1 (left panel), $p = 0.0328$; OVCAR5 vs OVCAR5/Pt, $p = 0.0012$; PEO1 vs PEO4, $p = 0.0015$; IGROV-1 vs IGROV-1/Pt1 (right panel), $p = 0.0218$, IGROV-1 vs IGROV-1/OHP, $p = 0.0384$.

A

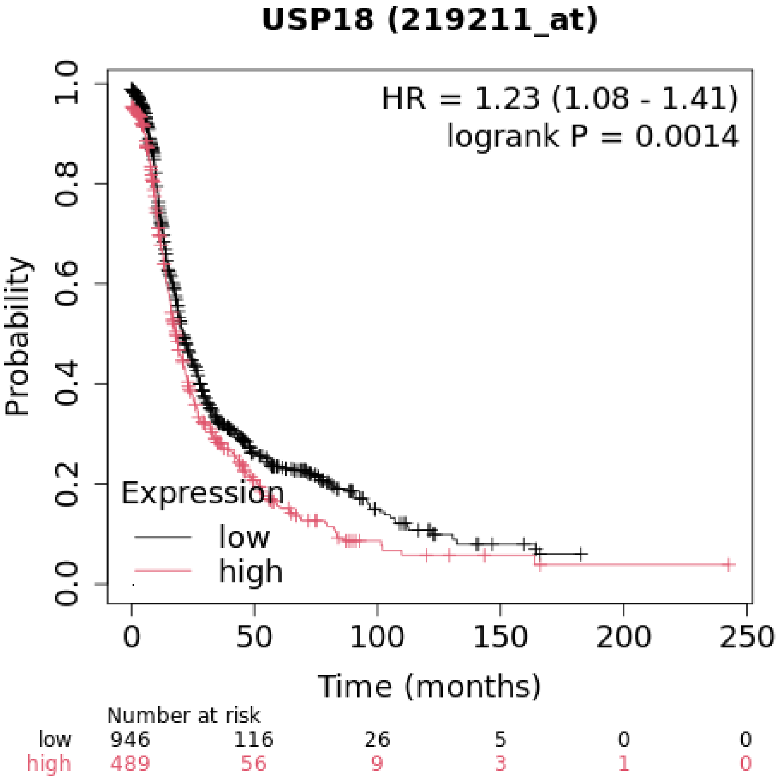

B

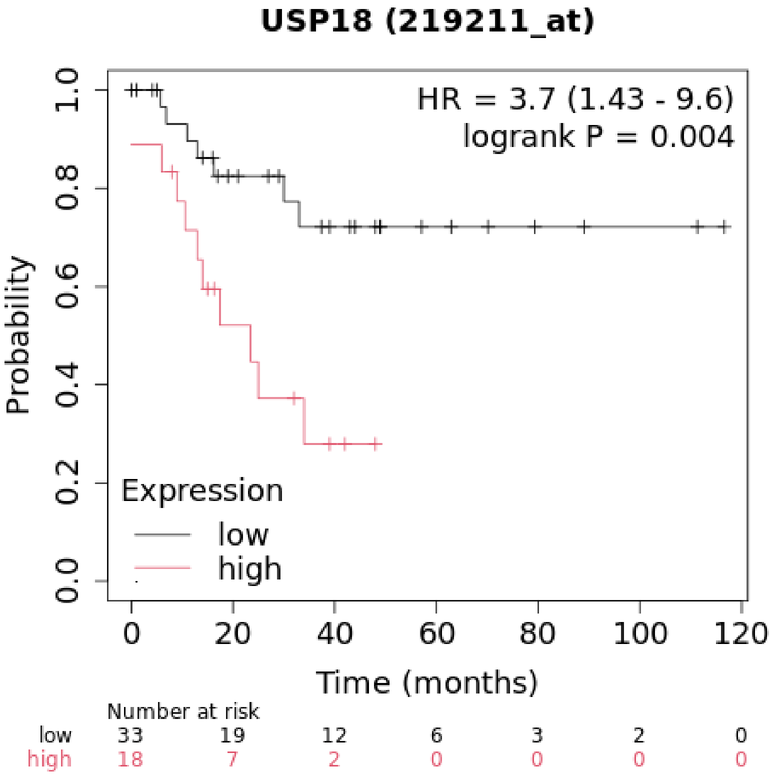

Supplementary Figure S4. Kaplan-Meier curves of progression free survival according to USP18 expression. The curves were obtained from the TCGA samples using KM plotter. (**A**) Samples from all ovarian carcinoma histological subtypes; (**B**) Samples from the endometrioid histological subtype.

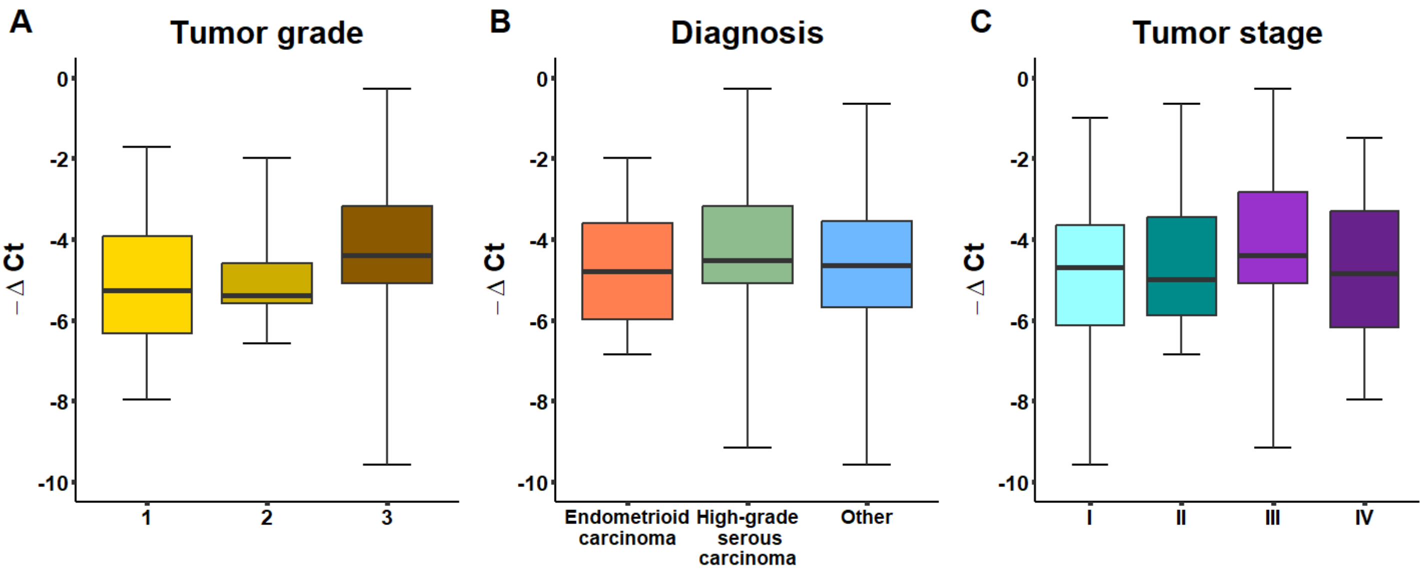

Supplementary Figure S5. USP18 relative expression distribution. Boxplot representing the expression of the USP18 (in terms of -ΔCt) according to tumor grade (A), diagnosis (B), and tumor stage (C). Each box indicates the 25th and 75th centiles of the distribution. The horizontal line inside the box indicates the median and the whiskers indicate the extreme measured values.

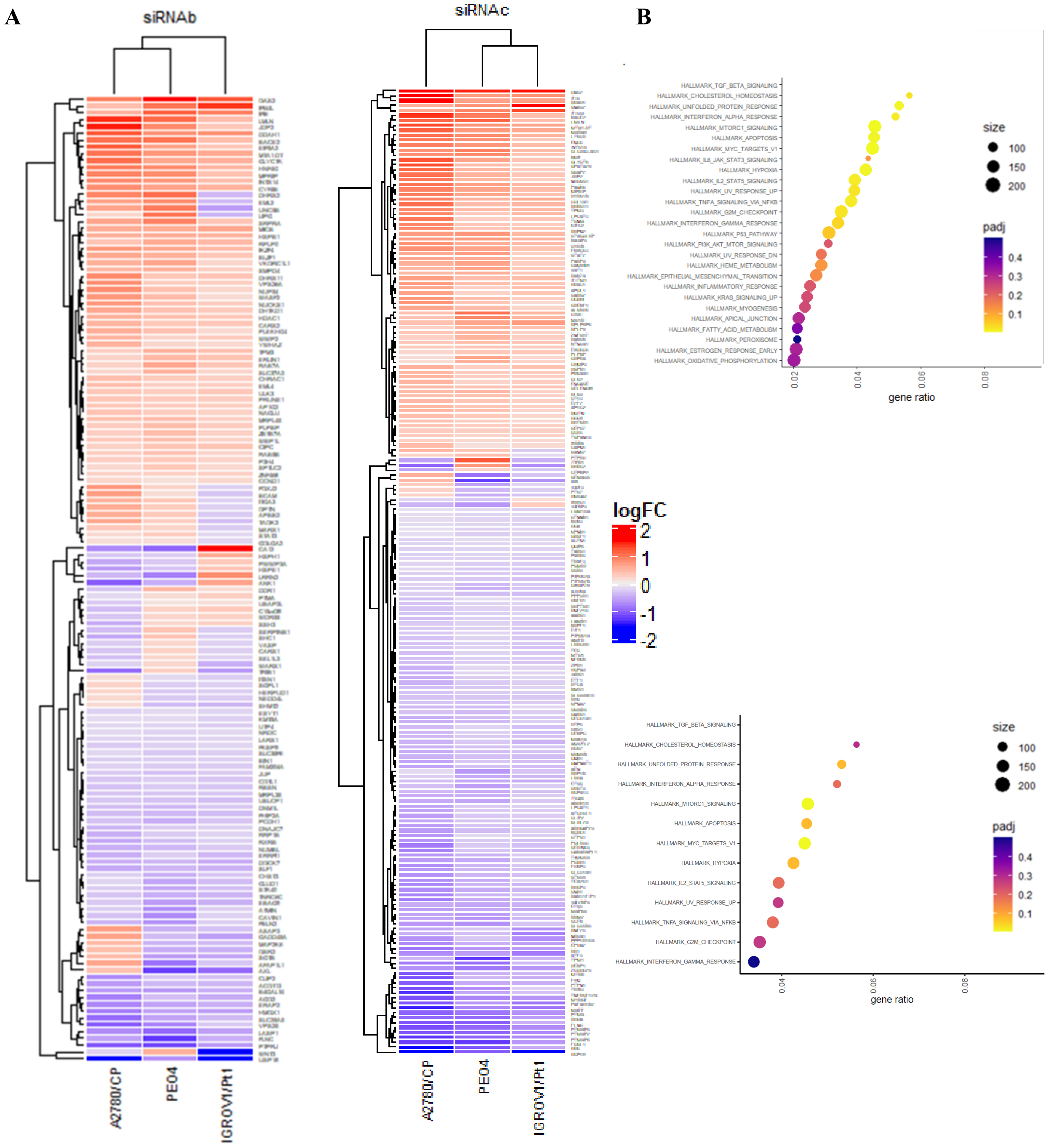

Supplementary Figure S6. RNA-seq analysis in different resistant ovarian carcinoma cell lines. (**A**) Heatmap of the fold change of the genes that are commonly differentially expressed in the 3 cell lines, depending on the siRNAs. Red boxes indicates a positive fold change; blue boxes a negative fold change. (**B**) Over Representation Analysis (ORA) on the Hallmark gene set collection for the genes that are commonly differentially expressed in the 3 cell lines depending on the siRNA.

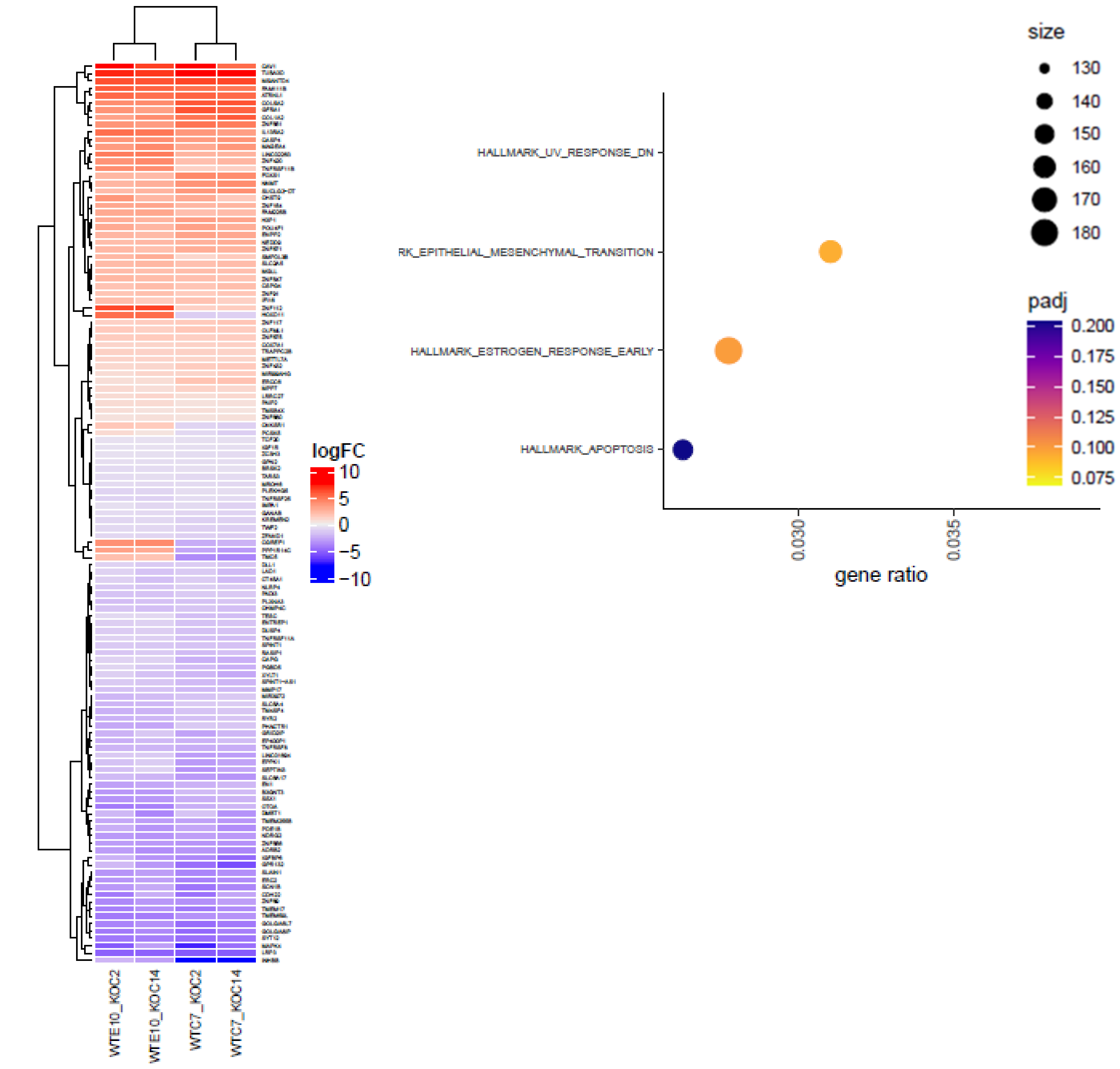

Supplementary Figure S7. RNA-seq analysis in CRISPR-Cas9 clones. (**A**) Heatmap of the fold change of the genes that are commonly differentially expressed in the 4 comparisons. Red boxes indicates a positive fold change; blue boxes a negative fold change. (**B**) Over Representation Analysis (ORA) on the Hallmark gene set collection for the genes that are commonly differentially expressed in the 4 comparisons.

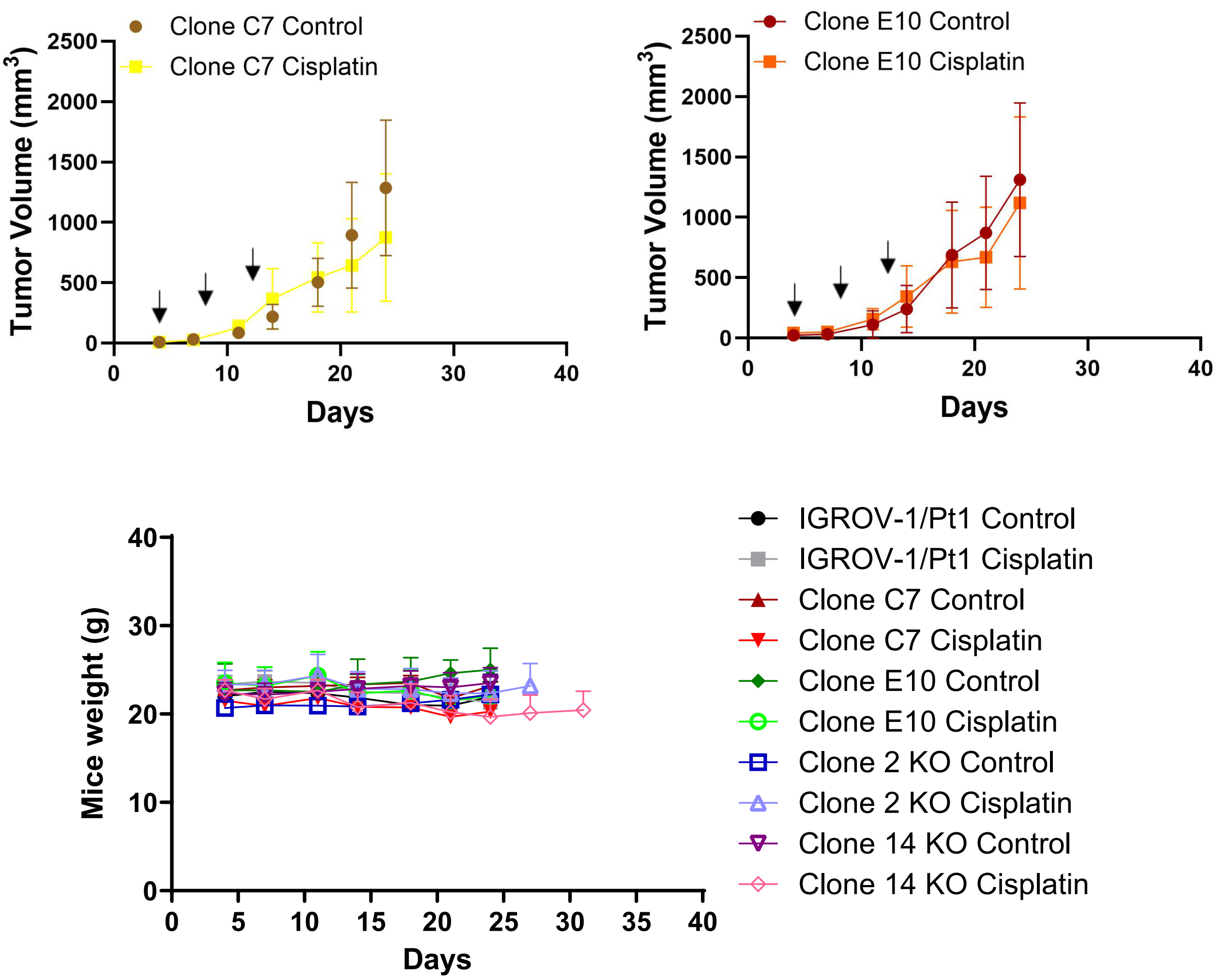

Supplementary Figure S8. Antitumor activity of cisplatin in athymic mice bearing human IGROV-1/Pt1-derived clones. Animals bearing tumors were treated by i.p route with 4.5 mg/kg cisplatin every week for 3 weeks. Treatment is shown by arrows. The antitumor activity is shown (A, B) together with mean mice weight over the experiment duration (C)

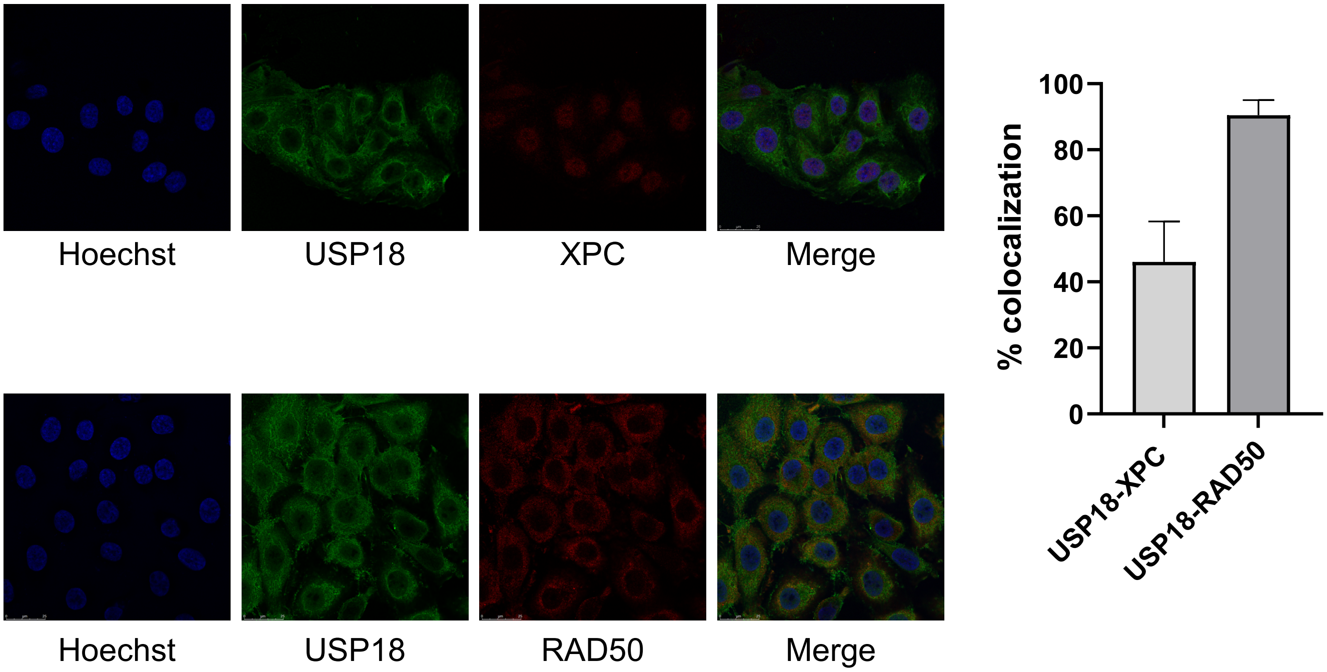

Supplementary Figure S9. Confocal microscopy in the parental IGROV-1/Pt1 cells. Representative confocal images of IGROV-1/Pt1 USP18/UBP43 cells with staining for USP18 (green), XPC or RAD50 (red), Hoechst (blue) and the merged image. The histogram indicates co-localization between USP18 and XPC or RAD50 proteins (mean ±SD of almost 3 different fields).

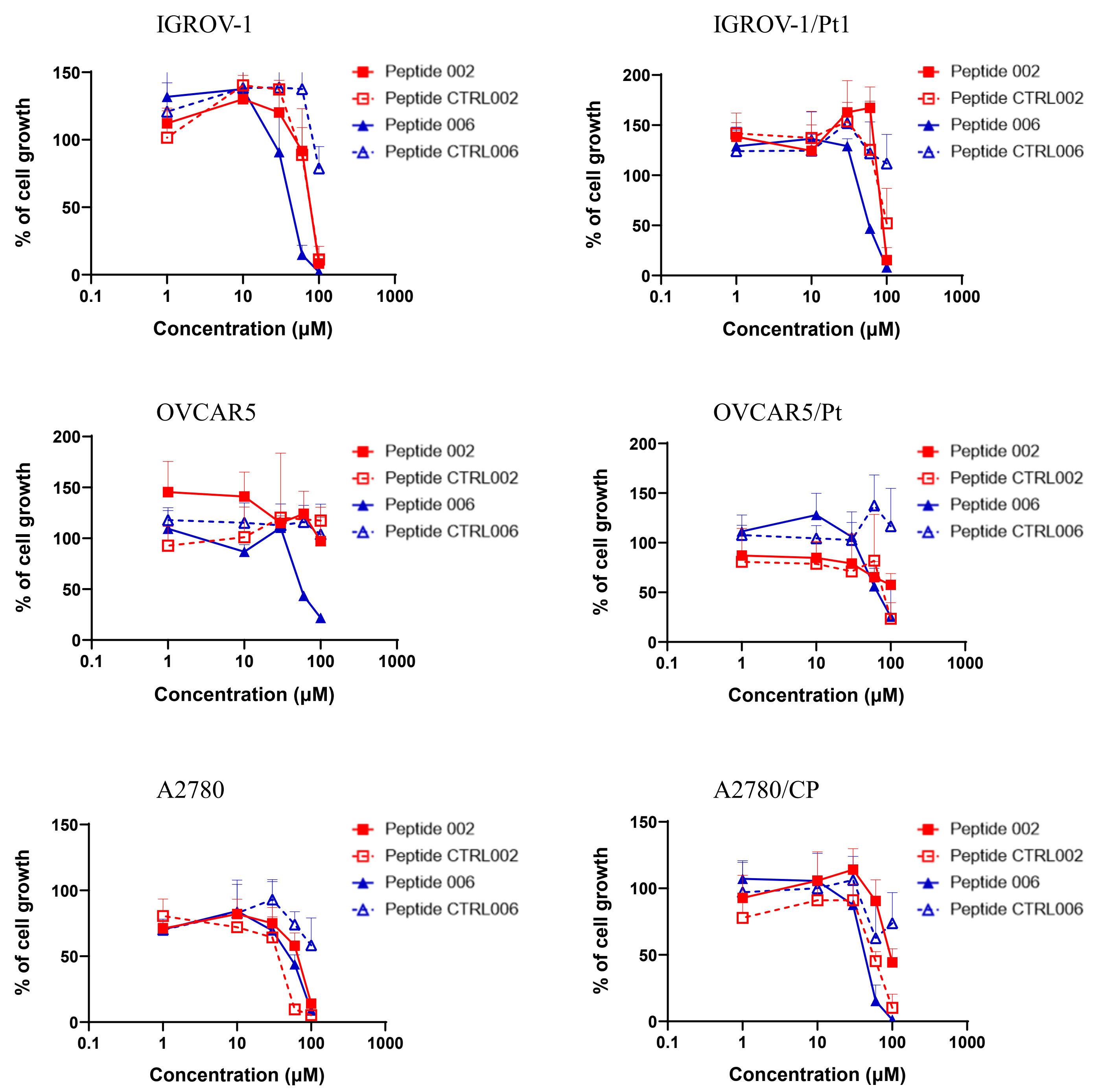

Supplementary Figure S10. Cell sensitivity to USP18-derived peptides in cisplatin-sensitive or –resistant ovarian cancer cell lines. Cells were seeded and 24 h later exposed to USP18-derived peptides (or relative control) in serum-free medium for 24 h; at the end of incubation the complete medium was added. Cell sensitivity was assayed 72 h after treatment start by MTS assay. The graphs reported mean ± SD of almost 2 independent experiments.

Table S4. **Sensitivity of ovarian carcinoma cells to USP18-derived peptides.**

	IC_{50} * (µM)					
**USP18 peptides**	IGROV-1	IGROV1/Pt1	OVCAR5	OVCAR5/Pt	A2780	A2780/CP
**Peptide 001**	>100	>100	>100	>100	>100	>100
**Peptide 002**	75	87	100	100	63	91
**Peptide CTRL002**	>100	>100	>100	75	33	55
**Peptide 003**	>100	>100	>100	>100	>100	>100
**Peptide 004**	>100	>100	>100	>100	>100	>100
**Peptide 005**	>100	>100	>100	>100	>100	>100
**Peptide 006**	41	57	53	66	49	42
**Peptide CTRL006**	>100	>100	>100	>100	>100	>100

* IC_{50} is the concentration inhibiting cell growth by 50% and it was calculated by the graphs (Supplementary Figure S10).
